# Experimental Investigation of Oxide Leaching Methods for Li Isotopes

**DOI:** 10.1111/ggr.12441

**Published:** 2022-07-20

**Authors:** Chun‐Yao Liu, Philip A. E. Pogge von Strandmann, Gary Tarbuck, David J. Wilson

**Affiliations:** ^1^ London Geochemistry and Isotope Centre (LOGIC) Institute of Earth and Planetary Sciences University College London and Birkbeck University of London Gower Street London WC1E 6BT UK; ^2^ Institute of Geosciences Johannes Gutenberg University 55122 Mainz Germany

**Keywords:** Li isotopes, oxides, sequential leaching, hydroxylamine hydrochloride, BCR‐2, SGR‐1b, Yellow River sediment

## Abstract

To examine the applicability of different leaching methods used to extract secondary oxides from silicate solids for lithium isotope (δ^7^Li) measurement, this study has conducted leaching experiments on five different types of silicate solids, including a fresh basalt, two weathered basalts, a Yellow River sediment (loess‐dominated) and a shale. Four factors were assessed in the experiments: the concentration of the leaching reagent hydroxylamine hydrochloride (HH), the leaching temperature (20 °C *vs* 95 °C), the leaching time and the reagent/solid ratio. Based on elemental concentrations and Li isotopes, 0.04 mol l^−1^ hydroxylamine hydrochloride (HH) in 25% *v/v* acetic acid at room temperature for 1 h with 40 ml g^−1^ reagent/solid ratio is recommended. At high temperatures, low δ^7^Li and high magnesium/iron ratios indicate that minerals other than secondary oxides are dissolved. With increased leaching time, there is no evidence for Li isotopic fractionation at room temperature. However, longer leaching time or increased reagent/solid ratios may increase the risk of leaching from non‐oxide phases. Meanwhile, results suggest that low concentrations of HH are not sufficient to target the secondary oxides evenly, while high concentrations of HH can leach out more non‐oxides. We also examined the optimal oxide leaching method within a full sequential leaching procedure (i.e., exchangeable, carbonate, oxide, clay and residual phases). Elemental concentrations show that no elements exist exclusively in oxides, so it is essential to analyse multi‐elemental concentrations to verify that the leaching has accessed this phase in a given sample. Comparing secondary oxides with their corresponding solutions, we estimate the isotopic fractionation (Δ^7^Li_oxide‐solution_) is −16.8‰ to −27.7‰.

## Key Points

- Different extraction methods for Li isotope ratios in oxides were examined.
- It is essential to determine element mass fractions in leachates to verify that the extraction is effective.
- We estimate that the isotopic fractionation between oxide and the corresponding solution (Δ^7^Li_oxide‐solution_) is −16.8‰ to −27.7‰.

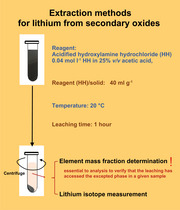

Chemical weathering, especially weathering of silicate rocks, is a fundamental control of the Earth's carbon cycle and hence climate, because weathering draws down atmospheric CO_2_, and significantly impacts ocean chemistry (Walker *et al*. 1981, Berner and Berner 1996, Gislason *et al*. 2009). In most weathering studies, the solids (i.e., primary minerals that dissolve during weathering, and secondary minerals such as clays and oxides that form during weathering) are analysed as bulk samples. However, since the pioneering method development of Tessier *et al*. (1979), the ability has existed to selectively extract (i.e., to leach) different phases within solids. While this approach has been widely used in studies of elemental behaviour, selective leaching is a relatively new field for methods that rely on “non‐traditional” stable metal isotopes.

These isotope systems have significantly increased in use over the past two decades, primarily due to the advent of second‐generation multi‐collector inductively coupled plasma‐mass spectrometers (MC‐ICP‐MS), and many previously unavailable metal isotopes systems (particularly light and transition metals) are now being used as tracers of geological processes (Teng *et al*. 2017). Of these systems, lithium (Li) isotopes have found particular interest in tracing weathering processes (Tomascak *et al*. 2016, Penniston‐Dorland *et al*. 2017, Pogge von Strandmann *et al*. 2020, 2021a). Lithium is the lightest metal element and has two natural stable isotopes, ^6^Li and ^7^Li (Dempster 1921, Meija *et al*. 2016a, b), with a relative mass difference of around 15%. Lithium is enriched in silicate solids over carbonates at the Earth's surface, such as river sediments and soils (Tomascak *et al*. 2016, Pogge von Strandmann *et al*. 2020). Due to the significant isotopic fractionation during weathering (e.g., up to 40‰ during clay formation), Li isotopes are regarded as one of the most promising tracers of silicate chemical weathering (Kisakürek *et al*. 2005, Misra and Froelich 2012, Pogge von Strandmann *et al*. 2020). During weathering, Li is dissolved from primary minerals without considerable fractionation (Wimpenny *et al*. 2010a, Pistiner and Henderson 2003), but then participates in secondary mineral formation. In secondary minerals, Li can be adsorbed (exchanged) on the negatively charged external and internal surfaces of the clays, as exchangeable Li (Velde 1995, Pistiner and Henderson 2003, Vigier *et al*. 2008, Wimpenny *et al*. 2015, Hindshaw *et al*. 2019, Pogge von Strandmann *et al*. 2020), while it can also enter the structural sites of clays during neoformation, largely by substituting for Mg (Vigier *et al*. 2008, Wimpenny *et al*. 2015, Hindshaw *et al*. 2019, Li and Liu 2020). Furthermore, Li can also be incorporated into oxides and oxyhydroxides, which are often considered together as the oxide phase (Chan and Hein 2007, Wimpenny *et al*. 2010b, Hindshaw *et al*. 2018). Most importantly, some studies have shown that the Li isotopic fractionation is closely related to the phases or the lattice sites where Li is taken up into. For example, there is relatively little Li isotope fractionation during uptake by the exchangeable pool, whereas Li uptake into octahedral sites is accompanied by considerable fractionation of up to 25‰ (Pistiner and Henderson 2003, Vigier *et al*. 2008, Wimpenny *et al*. 2015, Hindshaw *et al*. 2019, Li and Liu 2020, Pogge von Strandmann *et al*. 2020). It is therefore important to distinguish Li that is held in different solid phases and bonding environments to fully quantify both mass balance and weathering processes.

Two main groups of leaching methods have been applied to sediments and soils. One is the Tessier method (Tessier *et al*. 1979), and another is developed by the Community Bureau of Reference (BCR‐method) (Ure *et al*. 1993, Rauret *et al*. 1999). However, the applicability of a given leaching method varies according to the target geochemical tracers. Hence, modifications have been performed for specific tracers and different sample types based on these basic methods (Filgueiras *et al*. 2002), such as for Fe (Poulton and Canfield 2005), Mn (Lenstra *et al*. 2021), Zn (Fujiyoshi *et al*. 1994), Nd (Wilson *et al*. 2013) and Pb (Huang *et al*. 2021). To date, sequential leaching methods have only been sparsely applied and only partly developed to extract Li from different phases within silicate solids. The exchangeable Li is generally leached using sodium acetate (NaOAc) (Tessier *et al*. 1979, Hindshaw *et al*. 2018, Pogge von Strandmann *et al*. 2013, 2019a), acetic acid (HOAc) (Chan and Hein 2007, Wimpenny *et al*. 2010b) or ammonium chloride (NH_4_Cl) (Vigier *et al*. 2008, Hindshaw *et al*. 2019). Carbonate phases have very low Li contents and are generally considered not to affect the overall weathering behaviour of Li, which is therefore dominated by silicate weathering (Kisakürek *et al*. 2005, Dellinger *et al*. 2015), but this may not always be the case. Where explored, carbonate phases have been leached using HOAc‐buffered NaOAc or very weak acid (Pogge von Strandmann *et al*. 2013). Then, for the Fe/Mn/Al‐oxides/oxyhydroxides and clay phases, some studies have attempted to leach these phases together using HCl (Chan and Hein 2007, Wimpenny *et al*. 2010b, Pogge von Strandmann *et al*. 2013, 2019a). These HCl leachates were regarded as leaching either oxides or clays depending on which is the dominant phase in a given sample. For example, Wimpenny *et al*. (2010b) highlight the impact of the formation of Fe/Mn‐oxides/oxyhydroxides on Li isotopic fractionation, while Pogge von Strandmann *et al*. (2019a) emphasise the importance of clay formation. Finally, the residual phase is dissolved by strong acids, HF–HClO_4_–HNO_3_–HCl (Tessier *et al*. 1979, Pogge von Strandmann *et al*. 2013, 2019a).

As yet, it is not clear whether Li isotope behaviour is similar during oxide formation *versus* clay formation and if the potential multi bonding environment for Li in oxides (e.g., goethite, lepidocrocite and akageneite) could cause different Li isotope fractionation (Nielsen *et al*. 2005, Kim *et al*. 2008, Kim and Grey 2010, Wimpenny *et al*. 2015). Some research suggests that the formation of secondary Fe/Mn‐oxides/oxyhydroxides has a significant impact on Li isotopic fractionation (Vigier *et al*. 2008, Lemarchand *et al*. 2010, Wimpenny *et al*. 2010b, Hindshaw *et al*. 2018). In addition, dissolved Fe and Fe/Mn‐oxides/oxyhydroxides play important roles in the global carbon cycle and climate change in terms of both silicate weathering and organic carbon burial (Buck *et al*. 2007, Pogge von Strandmann *et al*. 2008, Jones *et al*. 2014, Hawley *et al*. 2017). To better constrain Li isotope fractionation during weathering, as well as to explore the significance of Fe/Mn oxide cycling, it is therefore essential to separate Li in Fe/Mn‐oxides/oxyhydroxides and clays as individual phases. Recently, Hindshaw *et al*. (2018) used 0.005 mol l^−1^ hydroxylamine hydrochloride (HH, NH₂OH·HCl) to target the secondary oxides contained in shales, which obtained significantly lighter Li isotope compositions for oxide phases than corresponding bulk samples. Furthermore, Li *et al*. (2020) used a more concentrated HH solution (0.5 mol l^−1^) to leach Fe/Mn‐oxides/oxyhydroxides within a Hawaiian soil profile. However, additional information (such as trace element concentrations) is not available from those studies, making it difficult to ascertain whether these extractions successfully targeted only the relevant phases, or also attacked other phases, although the methods are verified for other elemental or isotopic systems (Ure *et al*. 1993, Rauret *et al*. 1999, Wilson *et al*. 2013). Therefore, Li isotope fractionation factors for different phases remain poorly constrained.

This study has tested different oxide leaching methods applied to five different silicate solids, including a fresh basalt, two basaltic river sediments, a Yellow River sediment and a shale. In these experimental trials, the concentration of leaching reagent HH, leaching temperature, leaching time and the reagent/solid ratio were investigated as variables. Both Li isotope compositions and elemental concentrations (including Li) were analysed to assess whether such leaching methods are applicable to successfully extract Li from oxides and to inform recommendations for the most reliable leaching approaches.

## Materials and methods

### Materials

In this study, five silicate solids, including two rock reference materials that can be used to compare results for other studies, were used to examine the leaching methods targeting Li isotope measurements on the oxide phases. The solids were chosen to examine several different lithological types (i.e., fresh and weathered basalt, shale and loess), which would be expected to have different proportions and types of secondary minerals, including oxides. The fresh basalt used is the USGS reference material BCR‐2 (Basalt, Columbia River) (Wilson 1998). There are also two basaltic river sands (RS and RRS) from the Borgarfjörður estuary in west Iceland. This estuary has been highly studied for various isotope systems, including the isotopes of Li, Mg, Mo, Sr and U (Pogge von Strandmann *et al*. 2008, Pearce *et al*. 2010, Jones *et al*. 2014), and its sand has also been used in several basalt–water interaction experiments (Jones *et al*. 2012, Pogge von Strandmann *et al*. 2019a). One of the river sands examined here (RS) is the same as the solid used in those weathering experiments, and another river sand (RRS) is the reacted solid after the water‐rock interaction experiment of Pogge von Strandmann *et al*. (2019a). This experiment reacted the basalt sand with river water for around 9 months, with the formation of Mg smectites as well as other secondary phases occurring (Pogge von Strandmann *et al*. 2019a). The shale used is the USGS reference material SGR‐1b (Green River Shale) (Gladney and Roelandts 1988). The loess sample is sediment from the Yellow River (YR) estuary, to which loess from the Chinese Loess Plateau contributes more than 90% of the minerals (Zhang *et al*. 1990). This sediment was collected from the Ecological Tourism Area of the Yellow River Estuary (119°09′44.86″ E, 37°45′32.53″ N) on 6^th^ November 2019. The sample was dried in the oven at 60 °C after collecting. The elemental compositions for bulk samples of RS, BCR‐2, SGR‐1 and YR are given in Table 1, and their mineralogy is described in online supporting information Appendix S1.

**Table 1:** Element mass fractions in the bulk solids

Sample	Fe_total_	Mn	Al	Si	Ca	Mg	K	Li	Reference
	(mg g^−1^)	(μg g^−1^)	(mg g^−1^)	(mg g^−1^)	(mg g^−1^)	(mg g^−1^)	(mg g^−1^)	(μg g^−1^)	
RS^a^	101	2022	68	190	112	51	4	6	Jones *et al*. (2012)
BCR‐2	97	1520	71	253	51	22	15	9	Wilson (1998)
SGR‐1^b^	21	267	35	132	60	27	14	147	Gladney and Roelandts (1988)
YR	28	553	63	n.d.	43	13	19	28	This study^c^

n.d., not determined.

^a^ RRS is the reacted form of RS from an experiment in Pogge von Strandmann *et al*. (2019a). There is no discernible difference between RRS and RS in XRD and FTIR analysis (Appendix S1).

^b^ The element mass fractions of SGR‐1b are the same as SGR‐1 (Jochum *et al*. 2005, Wilhelms‐Dick *et al*. 2012).

^c^ Element composition of bulk YR was calculated from full sequential leaching in this study.

### Leaching methods and reagents

The oxide leaching methods examined here were conducted in the metal‐free clean laboratories of the London Geochemistry and Isotope Centre (LOGIC). A given mass of solids was weighed and added to tubes, followed by specific leaching reagents (see the following section; Tables 2 and 3). The tubes were agitated occasionally to make sure the solids and reagents remained well mixed. After each leaching step, samples were centrifuged at 8200 *g* (revolutions per minute) for 3 min in a Micro Star 12 Microcentrifuge (VWR®), and supernatants (i.e., leachates) were collected for further analysis or removed. The remaining solids after centrifugation were used for the next phase of leaching. The high‐temperature conditions (95 °C) were created with Pyrex water bath on a hot plate (ANALAB®), with a variability of 92–98 °C.

**Table 2:** List of oxide leaching method experimental trials

Sample	Solid	Reagent	Reagent/solid (ml g^−1^)	Time (h)	Temperature (°C)	Replicate samples
RS‐L1	RS	0.04 mol l^−1^ HH in 25% (*v/v*) HOAc	40	1	20	4
RS‐L3	RS	0.04 mol l^−1^ HH in 25% (*v/v*) HOAc	40	3	20	4
RS‐L6	RS	0.04 mol l^−1^ HH in 25% (*v/v*) HOAc	40	6	20	3
RS‐H1	RS	0.04 mol l^−1^ HH in 25% (*v/v*) HOAc	40	1	95	4
RS‐H3	RS	0.04 mol l^−1^ HH in 25% (*v/v*) HOAc	40	3	95	4
RS‐H6	RS	0.04 mol l^−1^ HH in 25% (*v/v*) HOAc	40	6	95	3
RS‐W	RS	0.005 mol l^−1^ HH in 2.6 mol l^−1^ HOAc	3	1	20	4
RS‐S	RS	0.5 mol l^−1^ HH in 0.05 mol l^−1^ HNO_3_ (pH = 1.5)	40	16	20	3
RRS‐L1	RRS	0.04 mol l^−1^ HH in 25% (*v/v*) HOAc	40	1	20	3
RRS‐L3	RRS	0.04 mol l^−1^ HH in 25% (*v/v*) HOAc	40	3	20	3
RRS‐L6	RRS	0.04 mol l^−1^ HH in 25% (*v/v*) HOAc	40	6	20	3
RRS‐H1	RRS	0.04 mol l^−1^ HH in 25% (*v/v*) HOAc	40	1	95	3
RRS‐H3	RRS	0.04 mol l^−1^ HH in 25% (*v/v*) HOAc	40	3	95	3
RRS‐H6	RRS	0.04 mol l^−1^ HH in 25% (*v/v*) HOAc	40	6	95	3
RRS‐W	RRS	0.005 mol l^−1^ HH in 2.6 mol l^−1^ HOAc	3	1	20	4
RRS‐S	RRS	0.5 mol l^−1^ HH in 0.05 mol l^−1^ HNO_3_ (pH = 1.5)	40	16	20	3
BCR‐L1	BCR‐2	0.04 mol l^−1^ HH in 25% (*v/v*) HOAc	40	1	20	4
BCR‐L3	BCR‐2	0.04 mol l^−1^ HH in 25% (*v/v*) HOAc	40	3	20	4
BCR‐L6	BCR‐2	0.04 mol l^−1^ HH in 25% (*v/v*) HOAc	40	6	20	4
BCR‐H1	BCR‐2	0.04 mol l^−1^ HH in 25% (*v/v*) HOAc	40	1	95	4
BCR‐H3	BCR‐2	0.04 mol l^−1^ HH in 25% (*v/v*) HOAc	40	3	95	4
BCR‐H6	BCR‐2	0.04 mol l^−1^ HH in 25% (*v/v*) HOAc	40	6	95	4
BCR‐W	BCR‐2	0.005 mol l^−1^ HH in 2.6 mol l^−1^ HOAc	3	1	20	4
BCR‐S	BCR‐2	0.5 mol l^−1^ HH in 0.05 mol l^−1^ HNO_3_ (pH = 1.5)	40	16	20	3
SGR‐L1	SGR‐1b	0.04 mol l^−1^ HH in 25% (*v/v*) HOAc	40	1	20	3
SGR‐L3	SGR‐1b	0.04 mol l^−1^ HH in 25% (*v/v*) HOAc	40	3	20	3
SGR‐L6	SGR‐1b	0.04 mol l^−1^ HH in 25% (*v/v*) HOAc	40	6	20	3
SGR‐H1	SGR‐1b	0.04 mol l^−1^ HH in 25% (*v/v*) HOAc	40	1	95	3
SGR‐H3	SGR‐1b	0.04 mol l^−1^ HH in 25% (*v/v*) HOAc	40	3	95	3
SGR‐H6	SGR‐1b	0.04 mol l^−1^ HH in 25% (*v/v*) HOAc	40	6	95	3
SGR‐L1‐V	SGR‐1b	0.04 mol l^−1^ HH in 25% (*v/v*) HOAc	167	1	20	4
SGR‐L3‐V	SGR‐1b	0.04 mol l^−1^ HH in 25% (*v/v*) HOAc	167	3	20	3
SGR‐L6‐V	SGR‐1b	0.04 mol l^−1^ HH in 25% (*v/v*) HOAc	167	6	20	3
SGR‐H1‐V	SGR‐1b	0.04 mol l^−1^ HH in 25% (*v/v*) HOAc	143	1	95	4
SGR‐H3‐V	SGR‐1b	0.04 mol l^−1^ HH in 25% (*v/v*) HOAc	143	3	95	4
SGR‐H6‐V	SGR‐1b	0.04 mol l^−1^ HH in 25% (*v/v*) HOAc	143	6	95	3
SGR‐W	SGR‐1b	0.005 mol l^−1^ HH in 2.6 mol l^−1^ HOAc	3	1	20	4
YR‐L1	YR	0.04 mol l^−1^ HH in 25% (*v/v*) HOAc	40	1	20	3
YR‐L3	YR	0.04 mol l^−1^ HH in 25% (*v/v*) HOAc	40	3	20	3
YR‐L6	YR	0.04 mol l^−1^ HH in 25% (*v/v*) HOAc	40	6	20	3
YR‐H1	YR	0.04 mol l^−1^ HH in 25% (*v/v*) HOAc	40	1	95	3
YR‐H3	YR	0.04 mol l^−1^ HH in 25% (*v/v*) HOAc	40	3	95	3
YR‐H6	YR	0.04 mol l^−1^ HH in 25% (*v/v*) HOAc	40	6	95	2

Grey shading indicates high temperature (95 °C) extractions.

**Table 3:** Element mass fractions and Li isotopes of sequential leachates

Sample	Solid	Target phase	Element mass fraction^a^							δ^7^Li^b^	2*s* ^b^
			Fe	Mn	Al	Ca	Mg	K	Li		
			(mg g^−1^)	(μg g^−1^)	(mg g^−1^)	(mg g^−1^)	(mg g^−1^)	(μg g^−1^)	(ng g^−1^)	(‰)	(‰)
RS‐ex‐1	RS	Exchangeable	0.00178	1.06	< 0.001	0.297	0.0726	57.7	43.7	10.46	0.31
RS‐ox‐1	RS	Oxides	1.10	63.7	1.17	0.990	0.469	22.6	82.6	3.27	0.16
RS‐clay‐1	RS	Clay	2.67	42.4	2.12	2.09	1.25	32.3	124	2.45	0.39
RS‐residue‐1&2^c^	RS	Residue	78.7	2010	70.8	75.7	47.5	2070	5030	1.23	0.15
RS‐bulk‐calculated‐1	RS	Bulk	82.5	2120	74.0	79.1	49.3	2180	5280	1.37	
RS‐ex‐2	RS	Exchangeable	0.0131	1.44	< 0.001	0.390	0.0863	37.2	30.7		
RS‐carb‐2	RS	Carbonate	0.0757	15.4	0.100	0.173	0.0425	27.0	30.3		
RS‐ox‐2	RS	Oxides	0.626	45.2	0.625	0.444	0.239	16.4	50.2		
RS‐clay‐2	RS	Clay	2.61	42.5	2.16	2.12	1.24	32.2	127		
RS‐residue‐1&2^c^	RS	Residue	78.7	2010	70.8	75.7	47.5	2070	5030	1.23	0.15
RS‐bulk‐calculated‐2	RS	Bulk	82.0	2120	73.6	78.8	49.1	2180	5260		
RS‐ex‐3	RS	Exchangeable	0.00551	1.84	0.00197	0.613	0.0954	46.3	48.1	11.58	0.22
RS‐carb‐3	RS	Carbonate	0.0727	13.7	0.0852	0.272	0.0473	22.3	51.6	6.92	0.37
RS‐ox‐3	RS	Oxides	0.860	51.0	0.770	0.574	0.328	12.9	69.9	2.18	0.39
RS‐clay‐3	RS	Clay	4.19	63.7	3.12	2.91	1.82	43.5	196	2.34	0.19
RS‐residue‐3	RS	Residue	79.5	1890	72.7	76.9	48.9	2220	4830	2.13	0.28
RS‐bulk‐calculated‐3	RS	Bulk	84.7	2020	76.7	81.2	51.2	2320	5190	2.28	
RS‐bulk‐reference^d^			101	2022	67.9	112	51.4	3700	5530		
BCR‐ex‐1	BCR‐2	Exchangeable	0.00189	0.834	<0.001	0.324	0.0984	135	19.2	2.81	0.26
BCR‐ox‐1	BCR‐2	Oxides	1.65	22.8	0.482	0.776	0.110	44.7	44.7	−2.01	0.10
BCR‐clay‐1	BCR‐2	Clay	3.62	43.8	0.400	2.42	0.510	22.7	206		
BCR‐residue‐1&2^c^	BCR‐2	Residue	90.7	1450	71.0	48.1	20.2	14300	8170	1.99	0.16
BCR‐bulk‐calculated‐1	BCR‐2	Bulk	96.0	1520	71.8	51.6	20.9	14500	8440		
BCR‐ex‐2	BCR‐2	Exchangeable	0.0210	1.05	< 0.001	0.422	0.107	136	28.8		
BCR‐carb‐2	BCR‐2	Carbonate	0.399	11.1	0.0440	0.153	0.0399	42.0	27.5		
BCR‐ox‐2	BCR‐2	Oxides	0.941	10.4	0.253	0.228	0.0484	33.1	26.6		
BCR‐clay‐2	BCR‐2	Clay	2.96	33.5	0.438	2.32	0.387	23.2	184		
BCR‐residue‐1&2^c^	BCR‐2	Residue	90.7	1460	71.0	48.1	20.2	14300	8170	1.99	0.16
BCR‐bulk‐calculated‐2	BCR‐2	Bulk	95.0	1510	71.7	51.2	20.8	14500	8440		
BCR‐ex‐3	BCR‐2	Exchangeable	0.00402	1.03	< 0.001	0.403	0.102	121	50.4	3.78	0.13
BCR‐carb‐3	BCR‐2	Carbonate	0.346	7.73	0.0271	0.151	0.0396	46.3	35.3	0.41	0.21
BCR‐ox‐3	BCR‐2	Oxides	1.07	9.46	0.268	0.296	0.0542	27.5	37.2	−1.86	0.19
BCR‐clay‐3	BCR‐2	Clay	5.04	60.6	0.535	2.86	0.660	23.4	320	1.16	0.21
BCR‐residue‐3	BCR‐2	Residue	93.0	1500	73.7	49.9	21.4	15400	7680	1.31	0.38
BCR‐bulk‐calculated‐3	BCR‐2	Bulk	99.4	1580	74.5	53.6	22.3	15600	8130	1.30	
BCR‐bulk‐reference^d^			96.5	1520	71.4	50.9	21.6	14900	9000		
YR‐ex	YR	Exchangeable	0.00518	1.08	< 0.001	1.15	0.202	101	133	5.23	0.05
YR‐carb	YR	Carbonate	0.0744	129	0.0297	26.6	0.378	75.8	170	6.75	0.34
YR‐ox	YR	Oxides	0.194	61.7	0.101	4.70	0.434	33.7	106	1.39	0.09
YR‐clay	YR	Clay	0.656	23.1	0.559	4.05	1.55	51.8	496	−1.66	0.08
YR‐residue	YR	Residue	27.0	338	62.8	7.13	10.5	18200	27500	0.73	0.39
YR‐bulk‐calculated	YR	Bulk	28.0	553	63.5	43.6	13.1	18500	28400	0.75	

^a^ Element mass fraction reports the mass of the element in the leachate per gram of solid leached.

^b^ δ^7^Li (with 2*s*) for each sample was measured by MC‐ICP‐MS three times (Section ‐ Lithium isotope and elemental determination).

^c^ The residue of RS‐1 and RS‐2 are mixed as one residue sample for elements and Li isotope measurement, and similarly for BCR.

^d^ The data of RS‐bulk‐reference and BCR‐bulk‐reference are from Wilson (1998) and Jones *et al*. (2012).

During the experimental procedures, all the PFA vials (Savillex), tips (Eppendorf®) and tubes were pre‐cleaned. The ultrapure water (resistivity = 18.2 MΩ cm) used was produced by a Merck Direct‐Q® 3 (Milli‐Q) system. The nitric acid (HNO_3_) was supplied as 68% *m/m* (AnalaR NORMAPUR, VWR Chemicals BDH®), and hydrochloric acid (HCl) was supplied as 32% *m/m* (AnalaR NORMAPUR, VWR Chemicals BDH®). Before use, both HNO_3_ and HCl were PTFE‐distilled using a DST‐1000 Acid Purification System (Savillex). The sodium acetate (NaOAc) solution was quantitatively dissolved from sodium acetate powder (AnalaR, VWR Chemicals BDH®) in ultrapure water. The hydroxylamine hydrochloride (HH) solution was quantitatively dissolved from hydroxylamine hydrochloride powder (99.999% trace metals basis, Sigma‐Aldrich®). The acetic acid (HOAc) was quantitatively diluted from glacial acetic acid (ROMIL‐SpA™ Super Purity Acids). Hydrofluoric acid (HF) was supplied as 47–51% *m/m* (ROMIL‐SpA™ Super Purity Acids) and perchloric acid (HClO_4_) as 65–71% *m/m* (ROMIL‐SpA™ Super Purity Acids).

### Leaching experimental trial design

Three different oxide‐leaching methods were tested, detailed in the following sections.

#### Tessier oxide leaching method

This leaching method is based on Tessier *et al*. (1979). Before oxide leaching, the exchangeable phases were removed using 0.8 ml 1 mol l^−1^ NaOAc at room temperature for 1 h (Pogge von Strandmann *et al*. 2019a). The oxide‐leaching reagent is 0.04 mol l^−1^ HH in 25% *v/v* HOAc. Based on this method, several oxide leaching experimental trials were designed to examine the influence of temperature, leaching time and the reagent/solid ratio (Table 2). For all five solid materials, around 50 mg solids were leached using 2 ml leaching reagents (i.e., a reagent/solid ratio of 40 ml g^−1^), at both room temperature (i.e., 20 ± 2 °C) and high temperature (i.e., 95 ± 3 °C) for 1, 3 and 6 h. For SGR‐1b, one more group of leaching was performed with a larger reagent/solid ratio (Table 2). Specifically, 12–14 mg for each solid were leached using 2 ml leaching reagent, giving a reagent/solid ratio of 143–167 ml g^−1^. The leaching was replicated three or four times for each specific leaching condition.

#### Weak oxide leaching method

This weaker leaching method is based on Hindshaw *et al*. (2018). In this study, for BCR‐2, RS, RRS and SGR‐1b, around 100 mg solids were leached using 0.3125 ml 0.005 mol l^−1^ HH in 2.6 mol l^−1^ HOAc (reagent/solid ratio of 3.125 ml g^−1^). The leaching was conducted at room temperature for 1 h. As above, the exchangeable phases were removed before oxide leaching. The leaching was replicated four times for each specific leaching condition.

#### Strong oxide leaching method

This stronger leaching method is from the Community Bureau of Reference (BCR, Rauret *et al*. 1999, Li *et al*. 2020), which recommends a stronger leaching solution of 0.5 mol l^−1^ HH in 0.05 mol l^−1^ HNO_3_ (pH = 1.5). In this study, around 50 mg of each sample of BCR‐2, RS and RRS were leached by 2 ml strong leaching reagent at room temperature for 16 h (reagent/solid ratio of 40 ml g^−1^). As above, the exchangeable phases were removed before oxide leaching. The leaching was replicated three times for each specific leaching condition.

Besides these oxide leaching experimental trials, based on Tessier *et al*. (1979), two full sequential extractions of RS and BCR‐2 and one full sequential extraction of YR were performed to inspect the interplay among the leaching phases. The full sequential extraction includes exchangeable, carbonate, oxide, secondary clay and residual phases in that order (Table 3). In addition, one sequential extraction of RS and BCR‐2 without a carbonate leach was also performed. For the exchangeable fraction, the solids were leached by 1 mol l^−1^ NaOAc at room temperature for 1 h. To target carbonate phases, 1 mol l^−1^ NaOAc buffered to pH 5 by HOAc was used at room temperature for 5 h (Pogge von Strandmann *et al*. 2019b, Tessier *et al*. 1979). The oxide leaching method was set at room temperature and the solids were leached by 0.04 mol l^−1^ HH in 25% *v/v* HOAc for 1 h. The clay phases were leached by 0.6 mol l^−1^ HCl at room temperature for 1 h. This clay leach is primarily designed to determine the isotope composition of secondary silicates, rather than to comprehensively remove them from the solids, and therefore significant clays will remain after this step and will be dissolved as part of the residue. Finally, the silicate residue was totally dissolved by HNO_3_ (∼ 68% *m/m*) – HF (47–51% *m/m*) – HClO_4_ (65–71% *m/m*), followed by HNO_3_ (∼ 68% *m/m*), and then 6 mol l^−1^ HCl (Pogge von Strandmann *et al*. 2019a).

### Lithium isotope and elemental determination

The Li isotopes of the leachates were measured by a multi‐collector ICP‐MS (Nu Plasma 3) after Li purification using a two cation‐exchange column method at the LOGIC laboratories (Pogge von Strandmann *et al*. 2019a). The cation‐exchange procedure used AG®50 W X‐12 resin and elution by 0.2 mol l^−1^ HCl. The details of the column procedure are in Table S2. Because Li isotopes fractionate during column elution, we monitored the column yield by collecting a split of the elution before and after the Li collection bracket, which were analysed for Li content. Most of the splits had < 0.1% of the total Li and < 0.3% of the total Li was present in all the splits. Thus, the shift in Li isotopes caused by incomplete recovery from columns was less than the long‐term precision in Li isotope ratio measurement (Gou *et al*. 2020, Wilson *et al*. 2021, Pogge von Strandmann *et al*. 2021a). For the Li isotope ratio measurements, a sample‐standard (calibrator) bracketing method was employed. The calibrator used was IRMM‐016, which has an isotopic ratio effectively identical to that of LSVEC (Jeffcoate *et al*. 2004, Pogge von Strandmann *et al*. 2019a). LSVEC is the delta‐zero reference material for Li isotopes, with ^7^Li/^6^Li = 12.17285 ± 0.00023, which is distributed by the International Atomic Energy Agency (IAEA) and National Institute of Standards and Technology (NIST) (Flesch *et al*. 1973, Qi *et al*. 1997, Magna *et al*. 2004). The sample solutions were nebulised using a CETAC Aridus II™ desolvating nebuliser system, ionised in the plasma torch, and sampled by a dry sampler cone (No. 319–646) followed by a skimmer cone that could enhance low mass elements (No. 301–020). In this instance, a 5 ng g^−1^ Li solution achieved a signal intensity of around 10 V (∼ 100 pA) of ^7^Li^+^, at an uptake rate of ∼100 μl min^−1^. The signal intensity of blank (2% *v/v* HNO_3_) was less than 0.03 V (0.3 pA). Each sample (*n* = 1) was measured a total of three times with ten ratios (50 s total integration time) for each time, and further details are reported in Pogge von Strandmann *et al*. (2019a). The Li isotope data are reported as the variation of the ^7^Li/^6^Li ratio in samples to the zero reference material LSVEC, shown in Equation (1):

(1) $$\delta^{7}\mathrm{Li}=\left(\left({}^{7}\mathrm{Li}{/}^{6}{\mathrm{Li}}_{\text{sample}}\right)/\left({}^{7}\mathrm{Li}{/}^{6}{\mathrm{Li}}_{\text{LSVEC}}\right)\right)-1$$

Atlantic seawater was analysed as “unknown” reference material, yielding a δ^7^Li value of 31.26 ± 0.54‰ (2*s*, *n* = 8), identical to published values (Jeffcoate *et al*. 2004, Pogge von Strandmann *et al*. 2019a, Gou *et al*. 2020). The long‐term measured value of Atlantic seawater at LOGIC is 31.17 ± 0.38‰ (2*s*, *n* = 43).

The element mass fractions of the leachates were determined by ICP‐AES (Varian 720) and ICP‐MS (Aglient 7900) instruments in the LOGIC laboratories. The mass fractions of Ca, Mg, K and Na for all phases were determined by ICP‐AES. The mass fractions of Fe, Al and Mn of the oxides, clays and residues were determined by ICP‐AES, while Fe, Al and Mn of the exchangeable and carbonate phases were determined by ICP‐MS. All Li mass fractions were determined by ICP‐MS. Leachates were diluted in 2% *v/v* HNO_3_ for ICP‐AES analysis first, and the solutions were divided into several matrices‐ (e.g., Na) matched groups for ICP‐MS analysis according to ICP‐AES results. The calibration lines for ICP‐AES and of each matrix‐matched group for ICP‐MS were constructed using a series of calibration standards made from ICP multi‐element standards or ICP single‐element standards, including TraceCERT® Periodic table mix 1 for ICP (Sigma‐Aldrich®), Instrument Calibration Standard 2 (SPEX CertiPrep®), Ca and Na (Agilent), Li and Mg (VWR® PROLABO®) and Fe, K and Al (PlasmaCAL, SCP SCIENCE). A standard (effectively the one most close to the most elemental concentrations of most samples) from the calibration standards were tested every ten to twenty samples as a drift monitor during each analysis batch. If the drift was larger than ±5%, the drift correction was applied. Two “external” reference solutions (i.e., solutions of dissolved bulk SGR‐1 and dissolved NBS SRM 88A) were analysed to assess accuracy and precision, and the results are within the recommended or published ranges (Gladney *et al*. 1987, Gladney and Roelandts 1988). The relative standard deviation (RSD) of individual analyses were better than ± 5%.

The elemental mass fractions in an extraction (a phase or “oxide” in a trial) from the total solid are reported as measured elemental mass fractions in leachates normalised to the total mass of the leached solid (see Appendix S3). An example of the mass fraction of Li in extraction A from the bulk solid is shown in Equation (2). The mass fraction of an element in an extraction (A) out of that element in the bulk solid is shown in Equation (3). In Equation (3), the values of [X]_bulk‐reference_ are the published values in Table 1.

(2) $$\;{\left[\mathrm{Li}\right]}_{A}\left(\text{mass fraction}\right)=\;\mathrm{Li}\;\text{mass concentration in the leachate of extraction}\;A\;\;\left(\text{from}\;\mathrm{ICP}‐\mathrm{AES}\;\text{or}\;\mathrm{ICP}‐\mathrm{MS}\right)\times \text{dilution factor}/\text{solid mass}$$

(3) $$F{\left[\mathrm{Li}\right]}_{A/\text{bulk}‐\mathrm{Li}}={\left[\mathrm{Li}\right]}_{A}/{\left[\mathrm{Li}\right]}_{\text{bulk}}$$

The reproducibility of the elemental mass fractions in the oxide leaching trials are shown as RSD in Table 4. Overall, the reproducibility of mass fractions and Li isotopes among replicates is fairly good (Appendix S2).

**Table 4:** Element mass fractions and Li isotopes of oxide leaching method experimental trials

Sample		Element mass fractions^a^														δ^7^Li	*s*
		Fe	% RSD	Mn	% RSD	Al	% RSD	Ca	% RSD	Mg	% RSD	K	% RSD	Li	% RSD	(‰)	(‰)
	*n*	(mg g^−1^)		(μg g^−1^)		(mg g^−1^)		(mg g^−1^)		(mg g^−1^)		(μg g^−1^)		(ng g^−1^)			
RS‐L1	4	1.16	4.9	73.6	3.8	1.22	4.9	0.984	4.3	0.516	5.8	20.4	7.2	92.0	2.2	3.67	0.42
RS‐L3	4	1.90	11.7	84.9	5.7	1.70	13.5	1.61	8.7	0.981	8.3	32.3	6.0	134	11.0	3.62	0.20
RS‐L6	3	2.51	3.9	90.6	4.5	2.03	4.7	1.85	5.0	1.21	4.0	30.9	8.9	159	3.4	3.46	0.37
RS‐H1	4	4.16	14.1	117	8.7	1.80	9.3	1.36	8.9	2.16	3.1	37.5	5.7	350	5.7	−1.74	0.21
RS‐H3	4	6.07	5.0	147	5.6	1.63	7.5	1.28	8.2	4.38	6.5	45.7	8.4	546	2.3	−2.10	0.14
RS‐H6	3	8.58	6.2	185	3.4	2.05	12.2	1.62	9.3	7.97	9.3	46.5	7.0	671	1.7	−1.47	0.48
RS‐W	4	0.338	36.4	38.5	21.6	0.362	34.3	0.278	38.7	0.124	41.0	6.47	23.6	30.0	24.4	5.59	0.73
RS‐S	3	5.25	7.1	124	7.8	3.90	7.6	5.15	5.9	2.98	7.3	68.1	5.9	396	31.7	2.36	0.30
RRS‐L1	3	1.30	7.6	69.2	2.5	1.25	7.8	1.08	9.6	0.542	9.7	20.7	9.1	123	2.5	5.33	0.61
RRS‐L3	3	2.02	3.5	79.3	2.1	1.66	3.8	1.61	2.3	0.943	3.4	27.6	1.0	146	3.4	5.87	0.16
RRS‐L6	3	2.79	8.0	89.0	6.1	2.16	7.0	2.16	6.9	1.36	6.3	40.3	11.3	277	7.4	5.74	0.56
RRS‐H1	3	7.05	4.0	148	3.8	2.49	5.9	2.36	5.5	5.21	4.4	47.0	9.4	480	5.6	0.74	0.32
RRS‐H3	3	9.71	2.6	192	3.8	2.07	9.8	2.17	4.0	8.98	3.3	41.6	5.0	699	3.6	0.03	0.78
RRS‐H6	3	11.9	9.3	222	7.3	1.91	1.2	2.45	10.8	12.4	9.3	46.9	39.6	802	10.5	0.89	0.57
RRS‐W	4	0.503	19.2	40.1	10.0	0.468	18.6	0.407	16.5	0.223	23.3	8.27	23.2	46.4	12.7	9.08	0.32
RRS‐S	3	6.01	12.5	136	8.4	4.34	11.3	4.75	11.6	3.78	12.6	62.9	5.6	312	10.0	3.67	0.19
BCR‐L1	4	1.26	4.1	17.7	4.1	0.345	9.6	0.758	8.1	0.091	7.0	37.7	9.3	39.1	5.9	−0.74	0.70
BCR‐L3	4	2.04	3.9	25.9	2.6	0.446	10.5	0.747	5.5	0.138	4.6	43.5	7.7	52.6	4.8	−1.33	0.71
BCR‐L6	4	2.54	4.3	31.3	5.3	0.509	4.1	0.699	5.0	0.184	3.3	42.4	4.1	69.3	9.5	−0.65	0.50
BCR‐H1	4	5.41	9.3	71.6	9.9	0.556	9.7	0.929	6.5	0.553	11.5	96.6	5.1	335	8.8	−0.94	0.13
BCR‐H3	4	8.34	2.2	119	2.1	0.540	2.8	0.826	2.5	1.08	2.7	130	2.6	630	7.0	−0.43	0.07
BCR‐H6	4	10.7	4.2	151	3.4	0.727	6.5	0.891	6.1	1.41	4.0	157	2.8	798	4.6	−0.42	0.05
BCR‐W	4	0.653	14.9	10.6	10.5	0.155	7.1	0.154	6.9	0.0490	19.3	20.2	12.4	20.2	12.9	−3.06	0.20
BCR‐S	3	4.59	2.4	51.3	2.4	0.801	1.5	4.34	0.4	0.472	3.6	50.0	8.9	164	5.8	−1.05	0.26
SGR‐L1	3	2.91	8.9	59.6	10.7	0.111	10.5	15.8	10.0	5.66	13.1	29.6	14.8	712	3.4	20.20	0.63
SGR‐L3	3	4.43	3.4	95.7	4.2	0.143	0.4	25.1	4.4	10.0	4.1	33.1	11.6	982	0.1	20.19	0.38
SGR‐L6	3	6.15	5.4	148	5.7	0.176	7.2	38.1	6.2	17.3	8.1	35.0	35.7	1500	0.6	20.64	0.46
SGR‐H1	3	7.86	3.1	182	3.8	0.217	5.4	46.8	4.8	21.7	4.4	77.6	2.3	2090	3.8	18.88	0.60
SGR‐H3	3	8.58	6.7	189	7.0	0.265	2.3	48.3	8.0	22.5	8.3	105	14.7	2290	3.8	16.58	0.29
SGR‐H6	3	8.72	7.9	193	5.3	0.274	7.3	48.7	5.8	22.9	6.2	116	5.1	2420	6.8	15.72	0.33
SGR‐L1‐V	4													1470	13.9	21.64	0.58
SGR‐L3‐V	3													1970	2.7	21.38	0.48
SGR‐L6‐V	3													2000	7.0	20.89	0.43
SGR‐H1‐V	4	7.98	6.7	204	5.8	0.304	7.4	45.3	7.0	21.3	5.5	97.0	9.5	2370	6.9	19.20	0.52
SGR‐H3‐V	4	8.10	5.9	190	6.4	0.364	5.1	41.3	6.3	19.7	6.0	163	10.1	3000	5.6	14.59	0.98
SGR‐H6‐V	3	8.27	17.2	169	15.0	0.444	13.9	36.6	12.2	17.6	14.4	224	14.4	4410	2.6	11.80	0.60
SGR‐W	4	0.96	3.6	18.5	3.1	0.0443	17.9	5.15	4.3	1.65	2.8	12.1	15.0	248	4.8	21.23	0.63
YR‐L1	3	0.25	7.1	211	4.2	0.172	3.7	36.9	4.7	0.715	7.7	36.0	28.9	212	5.5	5.06	0.18
YR‐L3	3	0.35	2.4	209	2.7	0.189	3.8	36.1	2.4	1.06	2.5	33.8	10.6	245	11.4	4.10	0.70
YR‐L6	3	0.48	2.7	228	4.6	0.233	1.4	39.6	5.3	1.44	1.6	43.2	24.0	277	1.7	3.85	0.88
YR‐H1	3	1.35	5.3	237	4.0	0.351	8.5	38.6	4.7	2.90	6.2	84.6	6.9	1210	1.5	−5.20	0.19
YR‐H3	3	2.06	2.4	249	1.6	0.562	5.2	38.9	1.3	3.22	0.5	96.9	4.5	2470	1.6	−4.67	0.39
YR‐H6	3	2.78	5.2	267	0.1	0.778	6.7	40.6	0.2	3.66	2.8	114	15.1	3700	6.9	−4.89	0.20

Grey shading indicates high temperature (95 °C) extractions.

^a^ Element mass fractions report the mass of the element in the leachate per gram of solid leached.

*n*, number of replicate.

## Results and discussion

### Factors in the Tessier oxide leaching method experimental trials

#### Temperature

**Figure 1:**
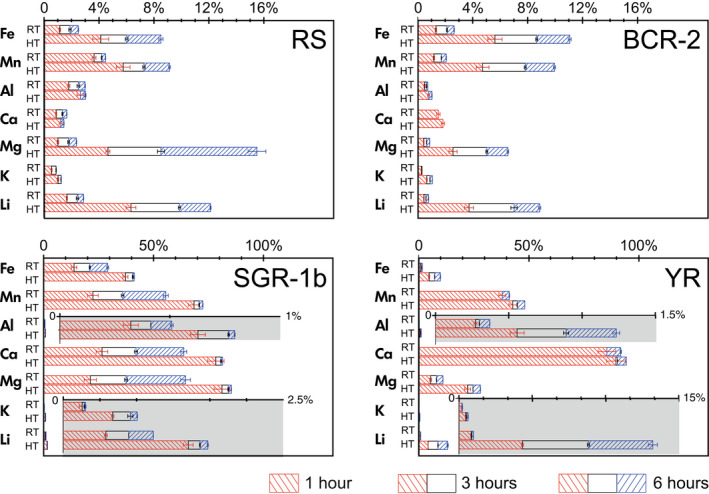
Elemental mass fractions in the experimental trials of the Tessier oxide leaching method as a percentage of the total elemental mass fractions of each bulk solid. RT, room temperature (20 ± 2 °C); HT, high temperature (95 ± 3 °C). The columns with separate axes in shaded areas are magnifications of data for Al, K and Li were required to better visualise the data. The range bar represents the standard deviation of the replicated samples. [Colour figure can be viewed at wileyonlinelibrary.com]

**Figure 2:**
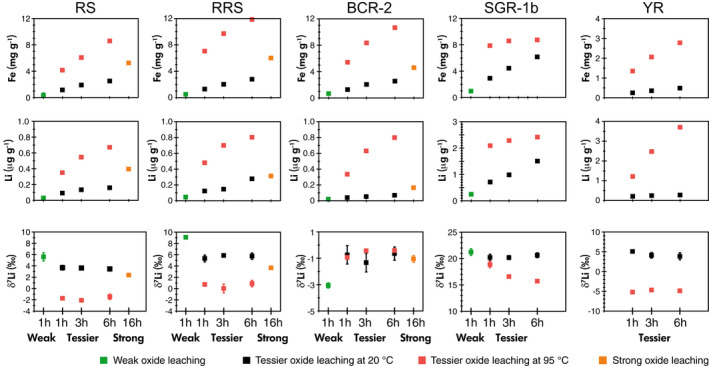
Iron and Li mass fractions and Li isotopes in oxide leachates from leaching experimental trials. See text for details of the different leaching methods. The range bars for Fe and Li mass fractions represent the RSD of the replicates, and the range bars for δ^7^Li represent the 1*s* of the replicates. The absence of visible range bars means that these bars are smaller than the symbol size. [Colour figure can be viewed at wileyonlinelibrary.com]

Leaching temperature has a significant impact on the leachate compositions (Table 4, Figure 1), which may indicate that different minerals are being attacked during oxide leaching at different temperatures. For example, as one of the major elements is expected to be high in the oxide phase, the Fe mass fraction is several times higher in the high‐temperature extractions than in the room‐temperature extractions for the same leaching time. This observation holds for all solid types: the Fe mass fraction is approximately twice as high for SGR‐1b at high temperature than at room temperature, while it is three times higher for RS, four times higher for BCR‐2, and five times higher for RRS and YR at high temperature than at room temperature, respectively (Figures 1 and 2). Chester and Hughes (1967) also showed that 25% HOAc‐acidified HH can dissolve more Fe at high temperature compared with room temperature during leaching of pelagic sediments, which they explained by the apparent targeting of clays.

Similarly, the extractions generally have enriched Mn mass fraction in higher temperatures, by approximately a factor of 2 in RS, RRS and SGR‐1b, and by a factor of ∼ 4 for BCR‐2, compared with room temperature leaching. However, in the YR leachates, there is no significant difference in the Mn mass fraction between high‐temperature and room‐temperature extractions (Figure 1). In the high‐temperature extraction, the mass fraction of Al, Ca and K are either similar to, or up to twice that in room temperature extractions when other leaching variables remain constant. Most notably, high‐temperature extraction increases the Mg mass fraction even more than that of Fe. The Mg mass fractions in the high‐temperature extractions are 1.5 to 4 times higher for SGR‐1b and YR, and four to ten times higher for RS, RRS and BCR‐2 than in the room temperature extractions (Figure 1).

The high‐temperature leaching also causes more Li to be leached out (Figures 1 and 2). For RS, RRS and SGR‐1b, the impact of high temperature on Li is similar to its impact on Fe, leading to around two to four times more Li being leached than at room temperature. For BCR‐2 and YR, the Li mass fraction increases even more significantly than Fe (Figure 2). Most interestingly, the δ^7^Li values in the leachates of RS, RRS, YR and SGR‐1b at high temperature for a range of leaching time (−1.8 ± 0.4‰ (*s*, *n* = 11), 0.6 ± 0.6‰ (*s*, *n* = 9), −4.9 ± 0.4‰ (*s*, *n* = 8) and 16.0 ± 2.6‰ (*s*, *n* = 20, also including different reagent/solid ratios), respectively) are distinctly lower than those from the room temperature leachates (3.6 ± 0.3‰ (*s*, *n* = 11), 5.6 ± 0.5‰ (*s*, *n* = 9), 4.3 ± 0.8‰ (*s*, *n* = 9) and 20.8 ± 0.7‰ (*s*, *n* = 19), respectively) (Figure 2). In contrast, the leachates of BCR‐2 do not show this difference and exhibit a narrow range of between −1.3‰ and −0.4‰, with a mean of −0.8 ± 0.5‰ (*s*, *n* = 24), in both room temperature and high temperature across a range of leaching time (Figure 2).

The elemental mass fractions and Li isotope data together imply that the targeted minerals or phases are different during high temperature and room temperature leaching. Generally, Fe in rocks has four forms: (a) bonded Fe in primary minerals (e.g., olivine, pyroxene) (Bigham *et al*. 2002, Jones *et al*. 2012), (b) primary Fe oxides (e.g., Fe‐Ti oxides and magnetite) (Bigham *et al*. 2002), (c) secondary clays (e.g., smectites) (Velde 1995) and (d) secondary Fe oxides/oxyhydroxides (e.g., goethite and lepidocrocite) (Bigham *et al*. 2002). Among these, only the secondary Fe oxides/oxyhydroxides that formed during continental weathering are the ones we aim to target here. Besides secondary Fe oxides/oxyhydroxides, the secondary oxides we aim to target also include secondary Mn oxides/oxyhydroxides (e.g., pyrolusite) and Al oxides/oxyhydroxides (e.g., gibbsite). During secondary mineral formation, Mn is preferentially incorporated into oxides/oxyhydroxides rather than into silicate secondary minerals such as clays. But it also tends to be included in carbonate and sulfide phases (see sections ‘Leaching time and Full sequential leaching’; Zhang *et al*. 1988, Lenstra *et al*. 2021). Gibbsite is the most common form of Al hydroxide and is widespread at the Earth's surface, while Al can also substitute for Fe in oxides/oxyhydroxides (Dixon and Weed 1989, Pinney and Morgan 2013). However, oxides/hydroxides are not the dominant phase for Al, because Al is present at considerable levels in clays and primary minerals. Moreover, gibbsite has similar features to clays, such as fine particle size and a capacity for the adsorption of cations (Velde 1995, Bowles 2021), and gibbsite can precipitate within the interlayer area of clays (Velde 1995). It is therefore difficult to reliably distinguish gibbsite from Fe/Mn‐oxides/oxyhydroxides phases or the clay phase. However, Ca, Mg and K are rarely contained within secondary oxides to any significant degree, so high mass fractions of those elements in an extraction targeting oxides would imply the influence of other phases (see Sections ‘Leaching time and Full sequential leaching’).

The reagent HH is a reducing agent, which is designed to reduce the transition metals, especially Fe and Mn. Theoretically, it can reduce Fe (III) to Fe (II) and Mn (III)/(IV) to Mn (II), as shown in Equations (4) and (5) (Bratsch 1989). Because both Fe(II) and Mn(II) are more soluble than in their oxidised states (Seidell 1919, Schwertmann 1991), these reactions enhance the leaching of oxides/oxyhydroxides. The reagent HH was used in 25% *v/v* HOAc (pH ≈ 2), leading to an acidic environment. Such an acidic environment can also play a role in leaching elements itself, such as Al as shown in Equation (6). Since Al is not a transition metal, Al‐oxides/oxyhydroxides are more sensitive to pH than pE (Eh), and it has been shown that gibbsite can be dissolved by dilute NaOH or HCl in a few minutes (Hashimoto and Jackson 1960, Hsu 1979, Dixon and Weed 1989). Importantly, the acidic conditions may also lead to partial leaching of other phases, such as carbonates, clays or other residual silicates.

(4) $$2{\mathrm{Fe}}^{3+}+2{\mathrm{NH}}_{3}{\mathrm{OH}}^{+}=2{\mathrm{Fe}}^{2+}+N_{2}+4H^{+}+2H_{2}O$$

(5) $$2{\mathrm{Mn}}^{3+}+2{\mathrm{NH}}_{3}{\mathrm{OH}}^{+}=2{\mathrm{Mn}}^{2+}+N_{2}+4H^{+}+2H_{2}O$$

(6) $$\mathrm{Al}{\left(\mathrm{OH}\right)}_{3}+3\text{HOAc}=3{\mathrm{Al}}^{3+}+3{\mathrm{OAc}}^{-}+3H_{2}O$$

It follows that temperature can affect elemental concentrations in the leachates by controlling the reductive ability of HH, the solubility of elements and the acidity of HOAc. According to standard electrode potentials (*E* ^*0*^) and their temperature coefficients, both the reductive ability of HH (N_2_(g)·H^+^/NH_3_OH^+^, *E* ^0^ = −1.83 V, d*E* ^0^/dT = −0.96 mV K^−1^) and the oxidative ability of Fe^3+^ (Fe^3+^/Fe^2+^, *E* ^0^ = 0.771 V, d*E* ^0^/d*T* = 1.175 mV K^−1^) and Mn^3+^ (Mn^3+^/Mn^2+^, *E* ^0^ = 1.56 V, d*E* ^*0*^/d*T* = 1.8 mV K^−1^) increase with temperature, with an approximately linear relationship in the range of 0–100 °C (Bratsch 1989). Generally, Fe in primary minerals exists as both ferrous and ferric Fe, while most of the Fe in secondary oxides is in a ferric form (Bigham *et al*. 2002). In addition, mineral crystallinity is an important factor. Amorphous and poorly crystalline Fe oxides are thought to be leached more readily than crystalline Fe oxides (Chao 1972, Gupta and Chen 1975, Chan and Hein 2007). So, theoretically, the order of Fe‐bearing minerals expected to be leached out would be secondary Fe oxide/oxyhydroxides, followed by primary Fe oxides, Fe‐bearing clays and then Fe‐bearing primary silicates. Similarly, the solubility of Fe and Mn in some aqueous solutions increases with temperature, as seen for FeCl_2_, FeCl_3_, Fe(NO_3_)_2_ and MnCl_2_ (Seidell 1919). The solubility of CaCl_2_, MgCl_2_, KCl and LiCl also increases with temperature (Seidell 1919). Therefore, the relatively high elemental concentrations in high‐temperature leachates may imply the dissolution of the minerals that are not explicitly targeted, such as primary oxides or clays. This hypothesis is supported by an experimental study on pyroxene dissolution by HCl–NH_4_Cl–NH_4_OH buffer solutions, which demonstrated that higher temperatures lead to increases in dissolution rates (Oelkers and Schott 2001). Another example is given by the dissolution of magnetite by oxalic, sulfuric and nitric acid, whereby dissolved Fe in the leachates at 50 °C is more than 10 times greater than in the leachates at 15 °C (Salmimies *et al*. 2011). Furthermore, the acid dissociation constant (*Kα*) of HOAc reaches a maximum at around 25 °C (Harned and Ehlers 1933). So theoretically, gibbsite may be leached more readily at room temperature than at high temperature. Further, the solubility of Ca(CH_3_COO)_2_ and Sr(CH_3_COO)_2_ slightly decreases with temperature from 10 to 100 °C (Seidell 1919, Apelblat 1993). In contrast, Mg(CH_3_COO)_2_ and K(CH_3_COO) become more soluble with increasing temperature (Seidell 1919, Apelblat 1993). Thus, the extremely high Mg mass fraction in the high‐temperature extractions is likely due to the partial dissolution of Mg‐rich clay or primary minerals. For example, the Mg mass fraction in the 6‐h oxide extraction of RS at high temperature represents 15% of the total Mg in the bulk RS (Figure 2). Since oxides rarely contain Mg, it is probable that another phase, likely the clay, is also undergoing leaching at the same time. Hence, it appears that secondary oxides do not always dominate the leach that is nominally designed to target the oxide phase, particularly when leaching is conducted at high temperatures.

In general, primary silicate minerals and phases have relatively low δ^7^Li values. For example, the δ^7^Li of the mantle is 3.5 ± 1.0‰ (Marschall *et al*. 2017) and that of primary basalt is 3–5‰ (Elliott *et al*. 2006). Similarly, the mean of the continental crust is 0.4 ± 0.4‰ (Sauzeat *et al*. 2015). In contrast, surface waters have high δ^7^Li values, with the global riverine mean being around 23‰ (Huh *et al*. 1998), and the δ^7^Li value of seawater is 31.2 ± 0.2‰ (Jeffcoate *et al*. 2004). Therefore, considering isotopic fractionation factors of approximately 10–20‰ for secondary mineral formation (Vigier *et al*. 2008, Wimpenny *et al*. 2015, Hindshaw *et al*. 2019, Li and Liu 2020), the secondary phases formed from these surface waters would generally be expected to be isotopically heavier than the primary silicates.

Given the above framework, the observation of consistently lower δ^7^Li values in the high‐temperature leachates than the room temperature leachates for all samples except BCR‐2 (Figure 2) appears to support the idea that phases such as igneous oxides or primary silicates, rather than only secondary oxides, are targeted at high temperatures. Since BCR‐2 is a basaltic igneous rock (Wilson 1998), its oxides are expected to be dominated by igneous oxides (Hamilton 1963, Flanagan 1967) (Appendix S1). The absence of any influence of temperature on the δ^7^Li values of the BCR‐2 leachates (Figure 2), which are as low as −0.8 ± 0.5% (*s*, *n* = 24), is consistent with the presence of only one primary oxide phase that is extracted at both low and high temperatures and suggests that igneous oxides are likely to have low δ^7^Li values. The other samples are a lacustrine sedimentary oil shale (SGR‐1b) (Boak and Poole 2015) and river sediments (RS, RRS and YR), all of which will have interacted with isotopically heavy surficial fluids and can be expected to contain two types of oxides, that are, primary igneous oxides and secondary oxides formed during chemical weathering (Appendix S1). Igneous oxides are present in the Icelandic basaltic river sands (Jones *et al*. 2012), and have also been reported as a constituent of the Yellow River loess (Pecsi 1990, Wang *et al*. 2009, Jin *et al*. 2021), while they may be less prominent in SGR‐1b (Appendix S1). Correspondingly, the leachates of RS, RRS and YR show strikingly different δ^7^Li values for high temperature and room temperature leaching (Figure 2), consistent with derivation from predominantly low‐δ^7^Li igneous oxides and high‐δ^7^Li sedimentary oxides, respectively. For the SGR‐1b leachates, the δ^7^Li values are high at ∼ 15–20‰ and are less sensitive to temperature, but they do shift towards lower values with increasing leaching time in the high‐temperature experiments, which is consistent with an increased contribution from isotopically light clay, igneous oxides or other primary silicate minerals. Furthermore, the full sequential leaching results from RS, BCR‐2 and YR conducted at low temperatures show that the residues, which are dominated by primary silicates, have δ^7^Li values higher than in the high temperature “oxide leachates” (see *Full sequential leaching*). Hence, the very low δ^7^Li values in the high‐temperature leachates likely arise from the preferential dissolution of a specific primary mineral, such as pyrite, Fe‐Ti oxides or pyroxene, which can have low δ^7^Li values (Penniston‐Dorland *et al*. 2017, Ionov and Seitz 2008, Tang *et al*. 2007).

#### Leaching time

In general, elemental concentrations in the leachates increase with leaching time. In the extraction of RS, RRS and BCR‐2, the increase in Fe mass fractions with time is more significant than increases in Mn, Al, Ca and K, but is similar to increases in the Mg mass fraction at room temperature, while at high temperature, Mg fractions increase more with time than Fe (Figure 1). The increases in Mg mass fraction may imply that more Mg‐rich minerals, such as from clays and/or primary silicates, are being leached out with increasing time, especially at high temperatures. Therefore, again, room temperature leaching is better than high temperature. Furthermore, at room temperature, the mass fraction of Fe and Li in the extraction by the Tessier method lay between the mass fractions from weak and strong oxide extractions (Figure 2). In other words, a more concentrated HH solution (0.5 mol l^−1^, acidified to pH = 1.5 by HNO_3_) employed for 16 h leaches out more Li and Fe than the Tessier leaching (0.04 mol l^−1^ HH, pH ≈ 2) does in 6 h. It suggests that the oxides may only be partly dissolved by the Tessier leaching method at room temperature, which has the advantage that the following phases in the leach cycle, clays and primary silicates, remain relatively unreacted. In addition, δ^7^Li in the leachates of RS, RRS and BCR‐2 are essentially constant through time, even while elemental mass fractions (including Li) increase with a longer leaching time (Figure 2). Hence, our experiments provide strong evidence that no significant Li isotopic fractionation occurs during the leaching of oxide phases by the reagent HH in HOAc. To be specific, neither Li re‐adsorption nor Li diffusion apparently occurred to any significant extent during these leaching experimental trials, which differs from recent dissolution experiments conducted on silicate rocks in weak acid in which Li fractionation during diffusion was suggested to have occurred (Verney‐Carron *et al*. 2011, Li *et al*. 2021). Importantly, due to the constancy of δ^7^Li through time, it is unlikely that the dominant mineral types in the leachates changed significantly over time. In other words, increasing leaching time leads to more removal of the oxide phase, but without a significant shift between isotopically heavy secondary minerals and isotopically light primary minerals. Overall, based on the results of RS, RRS and BCR‐2, the Tessier oxide leaching method for 1 h at room temperature is optimal, because the short time period is beneficial for minimising the possible risk of leaching from primary silicate minerals or clays, as well as saving time.

Unlike the leaching of RS, RRS and BCR‐2, the elemental mass fractions in the extractions of SGR‐1b and YR show some contrasting trends with leaching time. For SGR‐1b, except for K, most other elements (i.e., Fe, Mn, Al, Ca, Mg and Li) increase with leaching time at room temperature (Figure 1). The elemental mass ratios such as Mn/Fe, Ca/Fe and Mg/Fe also increase, while Al/Fe and K/Fe decrease with time (Figure S1). Somewhat unexpectedly, around 64% Ca, 65% Mg and 55% Mn out of the total amounts in the bulk solid are leached out after 6 h at room temperature (Figure 1). These numbers are around 26% Ca, 21% Mg and 22% Mn for the 1‐h room temperature leach and around 80% Ca, 81–85% Mg and 68–72% Mn for 1 to 6 h leaching at high temperature. These percentages in leachates of RS and BCR‐2 are only up to 2% Ca, 16% Mg and 10% Mn at high temperature for 6 h. These differences are likely linked to the differences in mineralogy (Appendix S1), and specifically the high carbonate content of SGR‐1b (Hamilton 1963, Jones *et al*. 2012, Boak and Poole 2015). Whereas BCR‐2 scarcely contains carbonates, and calcite accounts for only 4.3% of RS (Jones *et al*. 2012), calcite and dolomite could contribute more than 6% and 8% by weight, respectively, of the bulk SGR‐1b (Appendix S1). In addition, Mn and even Fe can also be incorporated in carbonates, such as in siderite and kutnohorite (Boak and Poole 2015). Given the acidity of the oxide leaching reagent (0.04 mol l^−1^ HH in 25% *v/v* HOAc, pH ≈ 2), it is enough to dissolve carbonates, which may therefore be included in the oxide leachates of SGR‐1b because no carbonate removal step was included before the oxide leaching. However, although the elemental concentrations suggest a carbonate influence on oxide leachates of SGR‐1b, the δ^7^Li in the room temperature leachates are consistent at 20.8 ± 0.7‰ (*s*, *n* = 19) through time (Figure 2). Presumably, either the [Li] in the carbonate is too low to influence the Li in the oxide leachates (Kisakürek *et al*. 2005, Dellinger *et al*. 2015), or the δ^7^Li values of the carbonate in SGR‐1b are similar to the oxides at around 20‰. At high temperature, δ^7^Li in the SGR‐1b leachates decrease through time (Figure 2), while the mass fractions of K, Al and Li, as well as ratios such as Al/Fe and K/Fe increase, while the mass fractions of Fe, Mn, Ca and Mg remain constant (Figures 1 and S1), which points to increasing dissolution of clays or primary minerals.

For the YR leachates, the increase in Fe mass fraction through time is more significant than for the other measured elements, so the mass ratios of other elements relative to Fe decrease with leaching time (Figure S1). In terms of mass fractions, elements in YR extractions at high‐temperature increase more significantly than extractions at room temperature with leaching time, especially Fe, Al, K and Li. Notably, the mass fraction of Mn and Ca in the YR oxide extractions are high and they remain relatively constant with leaching time (Figure 1). Around 85% Ca and 38% Mn out of the total in the bulk solids are leached out after 1 h at room temperature, increasing only slightly to 92% Ca and 41% Mn in the 6‐h leachates (Figure 1), consistent with the rapid dissolution of carbonates and/or Mn oxides (Wilson *et al*. 2013). The Yellow River estuarine sediments contain around 10% carbonate, and most of the carbonates are calcite from the Chinese Loess Plateau (Yang *et al*. 2009, Wang and Jin 2017) (Appendix S1), which appears to be readily leached. The full sequential leaching of YR in this study also supports that the oxide reagents (0.04 mol l^−1^ HH in 25% HOAc) can target carbonates (see Section ‘Full sequential leaching’). Correspondingly, δ^7^Li in the room temperature leachates slightly decreases from 5.1 ± 0.2‰ (*s*, *n* = 3) for 1 h to 3.8 ± 0.9‰ (*s*, *n* = 3) for 6 h. It seems therefore that carbonate does impact on δ^7^Li in the oxide leachates. Thus, for solids with a relatively high carbonate content, a carbonate removal step appears to be required before oxide leaching. In contrast, δ^7^Li in the high‐temperature leachates of YR is consistent at −4.9 ± 0.4‰ (*s*, *n* = 8), although the elemental concentrations increase over time. Thus, the continuous leaching of Li perhaps stems from a particular phase or mineral with a low δ^7^Li value.

#### Reagent/solid ratio

**Figure 3:**
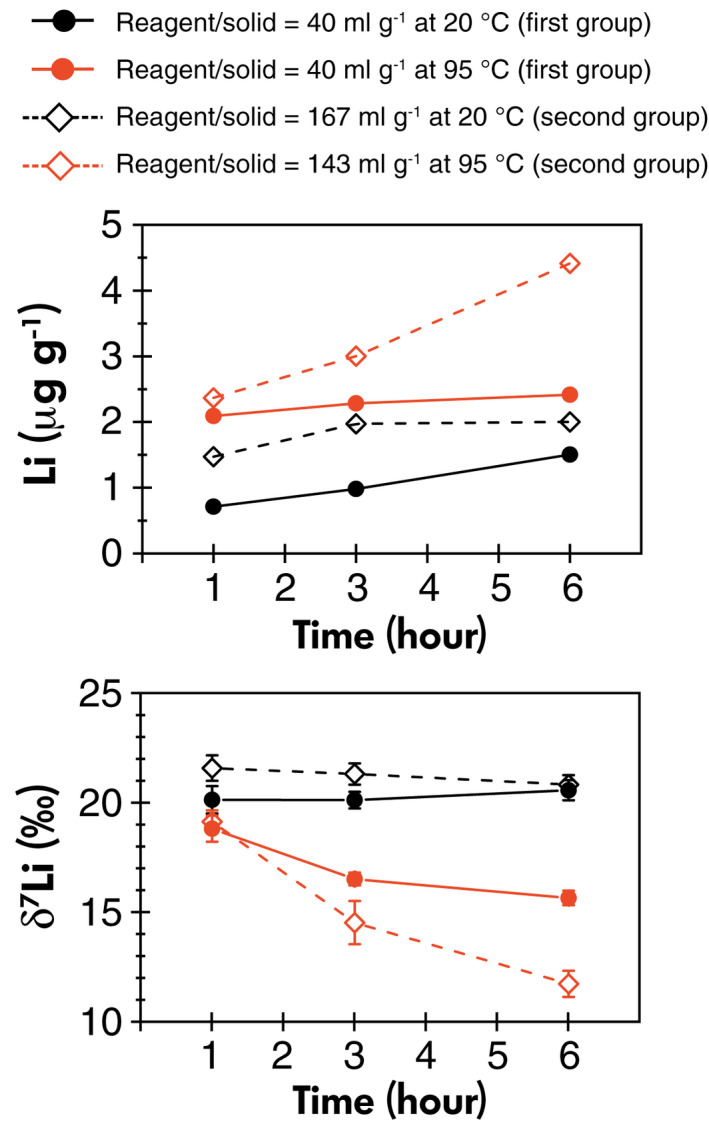
Lithium mass fractions and Li isotopes in oxide leachates of SGR‐1b using different reagent/solid ratios in Tessier oxide leaching experimental trials. [Colour figure can be viewed at wileyonlinelibrary.com]

For the SGR‐1b extraction, we also examined the impact of the reagent/solid ratio. In the first group, the reagent/solid ratio was 40 ml g^−1^, and in the second group, it was around four times higher at 167 ml g^−1^ (room temperature) or 143 ml g^−1^ (high temperature). Comparing the Li mass fraction in the low‐temperature extractions of the two groups shows that the second group (high reagent/solid) leaches out more Li per gram of solid than the first group (low reagent/solid) (Figure 3). After 1 h, the Li mass fraction in the second group of extractions is approximately two times higher than in the corresponding first group extractions, which provides evidence that the amount of leaching reagent is a limiting factor for Li in the first group, and therefore Li in oxide phases is only partly leached out. This has the advantage that it avoids attacking other phases. The δ^7^Li in the room temperature leachates in the first and second groups are virtually identical at 20.3 ± 0.5‰ (*s*, *n* = 9) and 21.3 ± 0.6‰ (*s*, *n* = 10), respectively (Figure 3). This consistency in the Li isotope compositions suggests that there is no Li isotopic fractionation and that the Li is probably derived from the same phase during leaching at room temperature from 1 to 6 h.

For the high‐temperature extractions, changes in the reagent/solid ratio do not lead to significant differences in the mass fractions of Fe and Mn (Table 4), whereas the mass fractions of K, Al and Li in second group extractions (high reagent/solid) are 1–2 times of that in the first group extractions (low reagent/solid). For Li, the mass fractions are similar for the two groups after 1 h but become more pronounced with time (Figure 3). Thus, it seems that 2 ml of reagent is sufficient to extract the majority of the oxides from ~14 mg solids during leaching at high temperature and that the use of a greater volume of reagents leads to more dissolution of clays or primary silicates. Correspondingly, the δ^7^Li in 1 h high‐temperature leachates of the first and second groups are similar at 18.9 ± 0.6‰ (*s*, *n* = 3) and 19.2 ± 0.5‰ (*s*, *n* = 3), while in 6 h leachates, δ^7^Li drops to 15.7 ± 0.3‰ (*s*, *n* = 3) in the first group and 11.8 ± 0.6‰ (*s*, *n* = 3) in the second group. Therefore, this supports the hypothesis that there is more influence by leaching of silicates (primary or secondary), which has a lower δ^7^Li, when there is excess leaching reagent than necessary for solely attacking the oxides. The excess reagent can lead to more leaching of silicates, which therefore dominate the Li stemming from the dissolution of the oxides.

### Comparison of Tessier, weak and strong oxide leaching methods

Besides leaching with 0.04 mol l^−1^ HH in 25% HOAc from the method of Tessier *et al*. (1979), this study examined other leaching methods on RS, RRS, BCR‐2 and SGR‐1b (Tables 2 and 4; Figure 2). One method is weaker leaching (Hindshaw *et al*. 2018), using 0.005 mol l^−1^ HH in 2.6 mol l^−1^ HOAc with a lower reagent/solid ratio (3.125 ml g^−1^). Another method is stronger leaching (Rauret *et al*. 1999, Li *et al*. 2020), using 0.5 mol l^−1^ HH in 0.05 mol l^−1^ HNO_3_ for a longer time of 16 h.

As expected, elemental mass fractions are lower with the weak oxide leaching method and higher with the strong oxide leaching method, when compared with the Tessier method at room temperature. For example, the Fe mass fraction leached by the weak oxide leaching method is only 20–50% of that by the Tessier 1 h leach, whereas the strong leachates have 3.6–4.6 times more Fe than the Tessier 1 h leach (Figure 2). The elemental stoichiometry also differs, with Mn/Fe and Al/Fe being higher in the weak leachates and lower in the strong leachates compared with the Tessier leach (Figure S1). According to their standard electrode potentials, Mn undergoes reduction more readily than Fe (Mn^3+^/Mn^2+^, *E* ^*0*^ = 1.56 V; Fe^3+^/Fe^2+^, *E* ^*0*^ = 0.771 V) (Bratsch 1989), which has also been observed in previous studies (Chester and Hughes 1967, Chao 1972, Gupta and Chen 1975). This agrees with the observed decreases in Mn/Fe with increasing leaching time (Figure S1). The slightly higher Al/Fe in the weak leachates may result from the high sensitivity of gibbsite solubility to pH (Hashimoto and Jackson 1960, Hsu 1979, Dixon and Weed 1989) and/or the decreasing stability of Fe oxides when Al substitutes for Fe in the oxides (Dixon and Weed 1989, Pinney and Morgan 2013). Thus, weaker leachates contain relatively more Mn oxides and other less structured oxides, and stronger leachates contain more Fe oxides and crystalline oxides.

Ratios such as Ca/Fe, Mg/Fe and K/Fe may provide clues on the influence of other phases, such as carbonate, clay and the exchangeable phases (Figure S1). For example, the Ca/Fe in the weak leachates of RS and RRS are similar to the Tessier room temperature leachates, while the weak leachates of BCR‐2 have lower Ca/Fe than the Tessier leaching (Figure S1). This may be due to the carbonate composition of the different solids. BCR‐2 scarcely contains carbonates, while calcite accounts for 4.3% of the Iceland river sand (Hamilton 1963, Jones *et al*. 2012). Further, if the continental silicates, that is, SGR‐1b and YR, contain over 10% carbonate, the carbonate phases may influence the δ^7^Li during oxide leaching (see Sections ‘Leaching time and Full sequential leaching’), given that the pH of the oxide leaching reagent is around 2, which is sufficiently low to dissolve carbonate. Likewise, the K/Fe in the weak leachates of BCR‐2 are nearly three times higher than in the strong leachates (Figure S1), due to the smaller amounts of Fe released during weak oxide leaching. Since exchangeable phases have significant amounts of K (see section ‘Full sequential leaching’), it may imply that some remaining exchangeable phases have an impact on the composition of those oxide leachates. Therefore, it is important to completely remove exchangeable and carbonate phases. Alternatively, strong oxide leaching methods (at room temperature) could be considered to increase the amount of oxide dissolution during oxide phase leaching, but meanwhile, it may increase the dissolution of primary oxides, clay or silicates. Furthermore, Ca/Fe and Mg/Fe are higher in the strong leachates of RS, RRS and BCR‐2 than in the Tessier room temperature leachates (Figure S1), which may indicate that more clays are attacked in these leaches due to longer leaching times and strong leaching acid. The lower δ^7^Li observed for the strong leachates compared with the Tessier leachates (Figure 2) is also consistent with increased contamination by other non‐oxide phases during strong oxide leaching.

**Figure 4:**
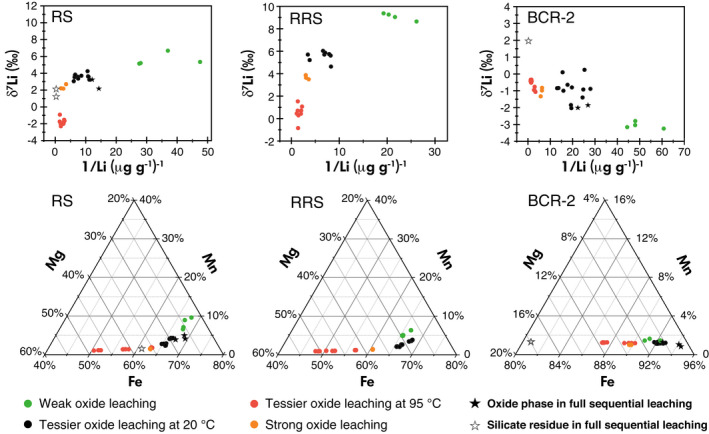
Lithium mass fraction – Li isotope diagrams and Mn–Mg–Fe ternary diagrams of oxide leaching trials of RS, RRS and BCR‐2. [Colour figure can be viewed at wileyonlinelibrary.com]

Based on the elemental mass fractions, theoretically, none of the leaching methods can ideally exclusively extract the secondary oxides as expected. In practice, the most optimal approach is making sure that Li from expected secondary oxides dominate the Li in oxide leachates. As discussed, the expected oxide phase here is the secondary oxides, including secondary Fe/Mn‐oxides/oxyhydroxides and secondary gibbsites (Al‐hydroxide). The potential interferences are silicates (including clays) and remaining exchangeable and carbonate phases. For RS and RRS, to a first‐order, the various oxide leaching methods appear to follow mixing trends for Li isotopes, except for the Tessier leachates at high temperature, which lie off those trends (Figure 4). As discussed earlier, the lower δ^7^Li values in the high‐temperature leachates are likely due to the additional dissolution of specific isotopically light minerals. For the other three oxide leaching methods of RS and RRS, the weak oxide extractions have the lowest Li mass fractions, which is only 35% of that of the Tessier room temperature extractions, and lie at the high δ^7^Li end of the mixing trend (Figure 4). Furthermore, the low mass fractions of Fe, Mg and Li, but high Mn/Fe and Al/Fe (Figures 2, 4 and S1) in these weak oxide extractions suggest that they have a greater contribution from “readily‐leached” oxides (i.e., less‐structured oxides and Mn oxides, and “pH‐sensitive” oxides (i.e., gibbsite)), which are considered to have higher δ^7^Li values (Chan and Hein 2007, Wimpenny *et al*. 2015). A study on marine ferromanganese deposits also showed that loosely bound Li has higher δ^7^Li than tightly bound Li within Fe–Mn oxides/oxyhydroxides (Chan and Hein 2007). The influence of exchangeable phases of RS and RRS, which have higher δ^7^Li, cannot be excluded, but the K/Fe in the weak leachates of RS and RRS are similar to that in the Tessier 1‐h room temperature leachates. Similarly, the weak leachates may contain some Li from silicates, but the low mass fractions of Fe and Mg suggest very little influence overall from silicates. So here, the weak leachates may be dominated by “readily‐leached” oxides with δ^7^Li as 5.6 ± 0.7‰ (*s*, *n* = 4) for RS and 9.1 ± 0.3‰ (*s*, *n* = 4) for RRS. The weak extraction, therefore, probably cannot dissolve all oxides evenly, although the weak extraction could reduce the influence of silicates. Meanwhile, the strong oxide extractions lie at the high Li mass fraction–low δ^7^Li end of the apparent mixing line, with around three times the Li mass fraction of the Tessier room temperature extractions. However, these leachates have high [Fe] and high Mg/Fe (Figures 2 and S1), which suggests a greater influence from (low δ^7^Li) silicates. The mass fraction of Mn in the strong extractions is lower than in the weak extractions and Tessier room temperature extractions, but similar to the silicate residues (Figure 4), which suggests that secondary Fe‐oxides, instead of “readily‐leached” oxides, dominate the oxide‐derived Li in the strong leachates. Therefore, it seems that the composition of the solutions stemming from strong oxide leaching is controlled by secondary Fe‐oxides, plus a degree of silicate material. The Li mass fractions and δ^7^Li in the Tessier room temperature leachates lie between the weak oxide leaching and the strong oxide leaching, which suggests that Tessier room temperature leachates consist of a mixture of both “readily‐leached” oxides (e.g., Mn‐ and Al‐oxides) and secondary Fe‐oxides. Although the influence of silicates on the Tessier room temperature oxide leachates cannot be excluded, their influence on these leachates is less than on the strong oxide leachates, as shown by lower Mg concentrations in the former. Furthermore, as discussed, although [Li] in Tessier room temperature leachates increases with time, the δ^7^Li remains constant (Figure 2). Hence, it is more likely that the Tessier room temperature leachates are closest to providing the composition of the “true” secondary oxide phases, which in turn are a mixture of “readily‐leached” oxides (i.e., Mn and Al oxides) and secondary Fe‐oxides, but fewer silicates.

For the leaching of BCR‐2, the weak oxide leaching shows the lowest δ^7^Li values of −3.1 ± 0.2‰ (*s*, *n* = 4), while the δ^7^Li in all the other leachates are −0.8 ± 0.5‰ (*s*, *n* = 27). As mentioned earlier, BCR‐2 is an unweathered igneous rock, whose oxide phases are isotopically the lightest among the five solids examined, while its clay (δ^7^Li = 1.2‰, where here there is likely little clay present, and this leach may also be targeting primary silicates) and residual phases (δ^7^Li = 1.8‰) are higher than for the “oxide leachates.” According to the elemental mass fractions, the bulk BCR‐2 has higher Fe/Mg than RS and RRS. The Fe–Mn–Mg ternary plot of the leachates of BCR‐2 shows very low Mn mass fractions, which also supports the idea that BCR‐2 has at most only trace amounts of secondary minerals formed at the Earth's surface. Given that the minerals in BCR‐2 formed at high temperatures (i.e., almost no low‐temperature secondary minerals exist), it is to be expected that isotope fractionation between the different phases is more muted than in the other samples that have all experienced low‐temperature weathering (Urey 1947).

### Full sequential leaching

**Figure 5:**
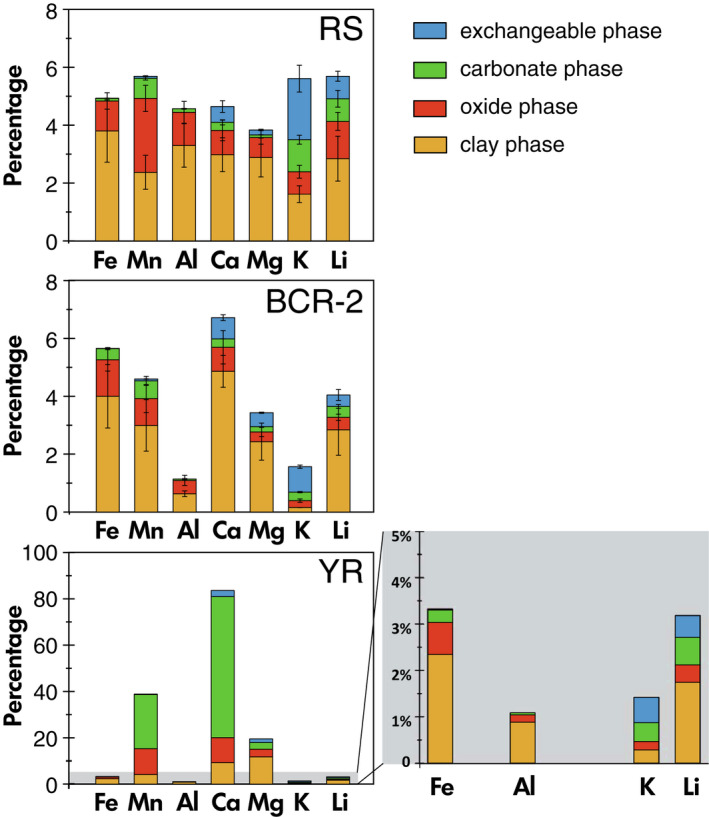
The mass fractions of elements in each phase, as a percentage of that in the bulk solid. The remainder of each sample is made up of the silicate residue (not plotted). [Colour figure can be viewed at wileyonlinelibrary.com]

The full sequential leaching method applied here is modified from Tessier *et al*. (1979), and comprises steps aiming to extract the exchangeable, carbonate, oxide, clay and silicate residues in the sequence. The leachates are regarded as corresponding operational fractions. The key point of such sequential leaching is to balance the influence of accidental leaching of the preceding and subsequent phases on the phase of interest. On the one hand, the targeted phase cannot be entirely leached out, because no leaching method is entirely efficient (Pogge von Strandmann *et al*. 2019a), and any attempt to achieve this would lead to other phases being attacked before the target phase is entirely dissolved. On the other hand, the incomplete leaching of a preceding phase may then contribute to the leachates of the following phase in the sequence (e.g., exchangeable phases in the carbonate phase, or carbonates in the oxide phase). Therefore, it is critical that the elemental composition of each phase is measured to assess contributions from other phases. In this study, partial leaching is employed to minimise the influence of the subsequent phase, because the Li concentrations generally increase with each phase in the sequence (Table 3, Figure 5).

The mass fraction of an element in each specific phase‐out of that element in a bulk solid can be calculated based on mass balance, with an example for Li in the oxide phase given as Equation (7) below. In addition, the bulk δ^7^Li values can be calculated based on the sum of the measured Li concentrations and δ^7^Li values for each phase in the sequence, as shown in Equation (8).

(7) $$\;F{\left[\mathrm{Li}\right]}_{\text{oxide}/\text{bulk}‐\mathrm{Li}‐\text{calculation}}={\left[\mathrm{Li}\right]}_{\text{oxide}}/{\left[\mathrm{Li}\right]}_{\text{bulk}‐\text{calculation}}\;={\left[\mathrm{Li}\right]}_{\text{oxide}}/({\left[\mathrm{Li}\right]}_{\text{exchangeable}}\;+{\left[\mathrm{Li}\right]}_{\text{carbonate}}+{\left[\mathrm{Li}\right]}_{\text{oxide}}\;+{\left[\mathrm{Li}\right]}_{\text{clay}}+{\left[\mathrm{Li}\right]}_{\text{residue}})$$

(8) $$\;\delta^{7}{\mathrm{Li}}_{\text{bulk}‐\text{calculation}}\times {\left[\mathrm{Li}\right]}_{\text{bulk}‐\text{calculation}}\;=\delta^{7}{\mathrm{Li}}_{\text{exchangeable}}\times {\left[\mathrm{Li}\right]}_{\text{exchangeable}}+\delta^{7}{\mathrm{Li}}_{\text{carbonate}}\times {\left[\mathrm{Li}\right]}_{\text{carbonate}}+\delta^{7}{\mathrm{Li}}_{\text{oxide}}\times {\left[\mathrm{Li}\right]}_{\text{oxide}}+\delta^{7}{\mathrm{Li}}_{\text{clay}}\times {\left[\mathrm{Li}\right]}_{\text{clay}}+\delta^{7}{\mathrm{Li}}_{\text{residue}}\times {\left[\mathrm{Li}\right]}_{\text{residue}}$$

By summing the elemental mass fraction from each leaching step, the bulk elemental mass fractions (i.e., Fe, Mn, Al, Ca, Mg, K and Li) of BCR‐2 are calculated as 90–105% of the corresponding published elemental mass fraction of BCR‐2 (Table 1; Wilson 1998). The calculated Mn, Mg and Li mass fractions of RS are 94–105% of the corresponding published elemental mass fractions of RS (Table 1; Jones *et al*. 2012). The calculated K, Ca, Fe and Al, which are consistent in three replicated full sequential extractions, are around 60%, 70%, 80% and 110%, respectively, of the published elemental mass fractions of RS (Table 1; Jones *et al*. 2012).

The Fe in the oxide leachates of RS, BCR‐2 and YR only comprises around 1% of the Fe in the bulk solids, which is less than in the clay phases and much less than in the silicate residue (which comprises primary and secondary silicates, including some clays) (Figure 5). It shows that Fe is included as a significant component in clays and primary silicates, and hence is not exclusive to oxides, although the oxide phases are only partly leached. Hence, as discussed in the ‘Temperature’ sub section, the Fe mass fractions in the nominal oxide leachates at high temperatures (around 8–11% of the total Fe in the 6‐h leachates of RS, BCR‐2 and YR; Figure 1) are excessively high. The elevated mass fraction suggests a significant degree of clay dissolution because the high temperatures can increase the reductive ability of HH, the solubility of elements in solution and the dissolution rates of minerals such as pyroxene and magnetite.

Among the five leaching phases of RS, BCR‐2 and YR, > 94% of the Li is contained in the silicate residues (Figure 5). The clay phase (i.e., HCl leach) is the second richest Li reservoir in RS, BCR‐2 and YR, comprising 2–4%. For BCR‐2, the oxide and carbonate phases represent similar Li reservoirs, at around 0.4%. In RS, oxides comprise 1–1.4% of the total Li, while carbonates comprise 0.6–1% Li. In YR, which has a greater carbonate content, slightly more Li is extracted in the carbonate phase than in the oxide phase, at around 0.6% and 0.35%, respectively. Generally, the exchangeable phases of RS, BCR‐2 and YR have similar Li mass fractions to their respective carbonate phases. Given that the Li budgets of the exchangeable, carbonate and oxide phases appear to be of a similar order of magnitude (Figure 5), it appears possible that these phases can influence each other during selective leaching. Thus, it is critical to analyse a suite of elemental concentrations in the leachates.

The sequential leaching also shows that Mg is a good indicator element for clays and primary silicates. For RS and BCR‐2, the clay phases contain around 4 and 7 times more Mg than the oxide phases, respectively, based on the average data of the replicated leachates, and residues comprise more than 95% of the Mg in the bulk solids (Figure 5). Furthermore, although the Yellow River sediments from near the estuary comprise 11% carbonates (Wang and Jin 2017), the carbonate leachates only contain around 2.9% of the total Mg in the bulk solid, reflecting the fact that most carbonates are relatively Mg‐poor calcite (Yang *et al*. 2009). Thus, Mg could be regarded as a reliable indicator for clays and primary silicates during leaching, even for the samples containing carbonate, so long as there is not a large proportion of dolomite. As discussed in the section entitled ‘Temperature’, in theory, Al can be found in several forms related to oxides, such as in gibbsite or substituting for Fe in oxides. Also, Al can occupy tetrahedral and octahedral positions in the clay lattice (Velde 1995), and Al is also a major element of certain igneous silicate minerals such as feldspar and mica. For RS, BCR‐2 and YR, Al in the residue comprises 95–99% of the Al in the bulk solids, while Al in the oxides comprises only 1% for RS, 0.3% for BCR‐2 and 0.16% for YR (Figure 5). The clay phase contains slightly more Al, at 3%, 0.6% and 0.9% of bulk RS, BCR‐2 and YR, respectively, although we note that the HCl leach will likely not remove the clays quantitatively because it is more designed to determine the clay composition rather than their elemental budget. For K, aside from the dominant residual phase, K evidently prefers the exchangeable phase to the other phases (i.e., carbonate, oxide and clay) (Figure 5). This finding is consistent with the proposal that K is more mobile and likely to be absorbed in the interlayer of clays rather than sitting in the bonded sites (Velde 1995, Gislason *et al*. 1996). Thus, K could be a useful indicator for exchangeable phases.

In general, carbonates are more prone to being leached than oxides or clay. Therefore, if a sample contains some carbonates, it is likely important to remove the carbonate phase before leaching oxides, especially for elements that are rich in carbonates, such as Ca, Mg, Mn or even Fe. For example, the YR carbonate leachates contain > 60% of the total Ca and around 23% of the total Mn in the bulk solid, with Mn/Ca as 4.86 mg g^−1^ (Figure 5). Meanwhile, the oxides only contribute around 11% of the total Mn. A previous study leached Yellow River sediment using the Tessier method, and demonstrated that more Mn was contained in the carbonate fraction compared with the oxide fraction (Zhang *et al*. 1988). However, a different study showed that some poorly ordered Mn oxides could be dissolved more readily than carbonate (Lenstra *et al*. 2021). In some published studies, the carbonates or calcite fossils that were targeted showed larger ranges of Mn/Ca, around from 0.1 μg g^−1^ to 35 mg g^−1^ (Misra and Froelich 2012, Pogge von Strandmann *et al*. 2013, 2019b, 2021b, Ullmann *et al*. 2013, Bastian *et al*. 2018, Washington *et al*. 2020, Dellinger *et al*. 2020). The high Mn/Ca stem from Mn‐carbonate during diagenesis (or from high Mn in seawater during reducing conditions, e.g., during Oceanic Anoxic Events) and/or contamination from Mn‐oxides. Thus, for YR, it is difficult to determine precisely how much Mn is in the oxide phase, as Mn could also stem from the carbonate phase. Mn therefore is not an indicator exclusively for oxides. Moreover, as indicated in the section ‘Leaching time’, if the oxide leaching of YR is conducted after removing only the exchangeable phase and not the carbonates, the leachates from the oxide step will include significantly more Ca and Mn as well as Li, likely from both carbonate and oxide. Furthermore, in some cases, sulfate or phosphate minerals (e.g., anhydrite and apatite) mineral could contribute minor Ca. In terms of Li isotopes, the carbonate phase in YR is sufficient to influence the δ^7^Li values in the oxide leachates if it is not removed before oxide leaching. Without removing carbonate, the δ^7^Li in the nominally oxide leachate is 5.1 ± 0.2‰ (*s*, *n* = 3), while the δ^7^Li in the oxide leachate after carbonate removal is 1.4‰. The δ^7^Li of the carbonate phase in YR is 6.8‰. As mentioned, the δ^7^Li of carbonate may be influenced by the Li isotopic composition in oxides. But, it generally falls within the reported range of the δ^7^Li value (−4.1 to 10.2‰) for carbonate in the Chinese Loess Plateau (Tsai *et al*. 2014, He *et al*. 2021, Xu *et al*. 2022). The elemental composition in the leachates of RS and BCR‐2 also indicates that oxide leaching could dissolve carbonates if they were not previously removed, but the impact on δ^7^Li is less than 1‰ (Figure 2 *cf*. 6).

**Figure 6:**
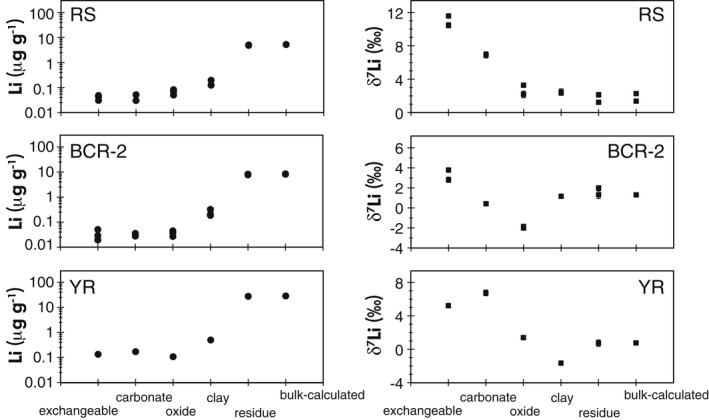
Lithium mass fractions and Li isotopes in each phase from the full sequential leaching for RS, BCR‐2 and YR.

The sequential leaching indicates that the different phases have different δ^7^Li and some consistent patterns emerge when comparing samples (Figure 6). For example, the exchangeable phases have higher δ^7^Li than oxides, clays and residues, especially for RS and YR. Their high δ^7^Li values may arise from the interaction with river water during weathering since both the riverine water in Borgarfjörður estuary (Pogge von Strandmann *et al*. 2008) and the Yellow River water (Gou *et al*. 2019) have δ^7^Li values of around 20‰. Thus, the fractionation between river water and exchangeable phases (Δ^7^Li_exchangeable‐solution_) is estimated to be −9‰ for RS and −15‰ for YR. Such values support the concept that the Li isotopic fractionation between surface waters and exchangeable phases is less than that between waters and structural sites in secondary clays, which is thought to be > 20‰ (Pistiner and Henderson 2003, Chan and Hein 2007, Vigier *et al*. 2008, Wimpenny *et al*.  2015, Hindshaw *et al*. 2019, Pogge von Strandmann *et al*. 2019a, Li and Liu 2020). The δ^7^Li in carbonate phases are 6.9‰ for RS, 6.8‰ for YR and 0.4‰ for BCR‐2 (Figure 6). Because BCR‐2 only has trace amounts of carbonate and has not undergone significant weathering, the δ^7^Li of its carbonate phase probably represents either primary carbonate, or an influence on the leach from exchangeable and oxide phases. In contrast, the δ^7^Li in carbonate fractions from RS and YR more likely reflect secondary carbonates, which would imply a fractionation between carbonate and the corresponding river water (Δ^7^Li_carbonate‐solution_) of around −13‰. This value is similar to, but slightly greater than, the published range of −8.5 ± 2‰ for inorganic calcite and −10.7 ± 0.5‰ for aragonite (Pogge von Strandmann *et al*. 2021a, Day *et al*. 2021). The clay and residual phases show relatively lighter Li isotopic compositions that range from −1.7‰ to 2.5‰ (Figure 6). The mass fraction of Li in the clay phase is two times greater than the amount in the oxide fraction for RS, 8 times greater for BCR‐2 and 5 times greater for YR, so the δ^7^Li of the clay phases is unlikely to be significantly affected by Li from oxides. Residual phases contribute more than 90% of the Li in the bulk sample, which is also unlikely to be influenced by remaining Li from preceding phases, and this residual phase dominates the calculated composition of the bulk sediments. The calculated bulk δ^7^Li values are around 2.0‰ for RS and 1.6‰ for BCR‐2, which are slightly lower than the published bulk δ^7^Li values of 4.4‰ for RS (Pogge von Strandmann *et al*. 2019a) and 2.6–3.5‰ for BCR‐2 (Rosner *et al*. 2007, Pogge von Strandmann *et al*. 2011, 2012, 2019a, b, Penniston‐Dorland *et al*. 2012, Choi *et al*. 2013, Genske *et al*. 2014, Ryu *et al*. 2014, Liu *et al*. 2015, Bohlin *et al*. 2018, Liu and Li 2019, Li *et al*. 2019, 2020). The calculated bulk δ^7^Li for YR of 0.8‰ is consistent with the published δ^7^Li values for bulk loess from the Chinese Loess Plateau and Yellow River sediments, which range from 0.6‰ to 6.9‰ (Tsai *et al*. 2014, Gou *et al*. 2019, He *et al*. 2021, Xu *et al*. 2022).

### Implications for Li isotopic fractionation by oxides during weathering

The published δ^7^Li values for oxide phases exhibit a wide range (Table 5), from −14.5‰ to 32.8‰ (Chan and Hein 2007, Wimpenny *et al*. 2010b, Hindshaw *et al*. 2019, Li *et al*. 2020). We note, however, that most of these studies did not publish elemental concentrations of their leachates and did not necessarily rigorously investigate the consequences of different leaching methods. Therefore, it is possible that these data on nominal oxide leachates could also in fact include fractions of other phases. Based on the published δ^7^Li values for oxides and their corresponding solution δ^7^Li, the Li isotopic fractionation factor (*α*_oxide‐solution_) is observed to vary from 0.972 to 1.001 (Table 5). This wide range spans the published fractionation factors between aqueous solutions and a wide range of potential solid phases, including the exchangeable phase, secondary clay phase and bulk solids (Pogge von Strandmann *et al*. 2020). Here, we note that some *α*_oxide‐solution_ values, which are calculated based on published data, are difficult to confirm as equilibrium Li isotopic fractionation factors between oxide and solution, especially in Hawaii and Svalbard (Hindshaw *et al*. 2019, Li *et al*. 2020). It is also likely that the Li isotopic fractionation between solutions and oxides in natural samples is smaller at higher temperatures (according to stable isotope fractionation theory, Urey 1947), which is confirmed by observations (Vigier *et al*. 2008, Hoefs 2009, Li and West 2014, Gou *et al*. 2019, Pogge von Strandmann *et al*. 2021a). For example, the *α*_oxide‐solution_ for tropical Hawaiian soil ranges from 0.991 to 0.994, while *α*_oxide‐solution_ in the Borgarfjörður estuary in Iceland, in the rivers in Greenland and Svalbard, and in the Yellow River are within the range of 0.979–0.983 (Table 5) (Wimpenny *et al*. 2010b, Hindshaw *et al*. 2019, Pogge von Strandmann *et al*. 2019a, Li *et al*. 2020). Given the wide range of reported isotopic fractionations for oxides (Table 5), we suggest that Li isotopic geochemical behaviour during secondary oxides formation should now be revisited with the application of optimised oxide leaching methods. Material characterisation techniques and chemical structure analysis, such as scanning electron microscopy and solid‐state nuclear magnetic resonance, could be applied to explore the Li fractionation in different oxide minerals and different bonding environments in the future.

**Table 5:** Lithium isotopic fractionation between solutions and oxide phases

Sample	Solution δ^7^Li (‰)	Oxide δ^7^Li (‰)	Oxide Li (ng g^−1^)	Δ^7^Li_oxide‐solution_ (‰)	*α*_oxide‐solution_	Leaching method
RS	20	3.2	70	−16.8	0.983	0.04 mol l^−1^ HH in 25% *v/v* HOAc, room temperature, 1 h
RRS	33	5.3	120	−27.7	0.972	0.04 mol l^−1^ HH in 25% *v/v* HOAc, room temperature, 1 h
YR	19.4	1.4	106	−18.0	0.982	0.04 mol l^−1^ HH in 25% *v/v* HOAc, room temperature, 1 h
BCR‐2		−1.5	40			0.04 mol l^−1^ HH in 25% *v/v* HOAc, room temperature, 1 h
SGR‐1b		20				0.04 mol l^−1^ HH in 25% *v/v* HOAc, room temperature, 1 h
Greenland river suspended load	26	5	18000	−21	0.979	2 mol l^−1^ HCl
Dryadbreen glacial sediment	10	−7.5	160	−17.5	0.983	0.005 mol l^−1^ HH in 2.6 mol l^−1^ HOAc
Hawaii soil	20	11.4–14.1	445–1499	−8.6 to −5.9	0.991–0.994	0.5 mol l^−1^ HH in 0.05 mol l^−1^ HNO_3_ (pH = 1.5)
Ferromanganese (hydrogentic)	28–32	13.4–28.2	640–10100	−18.6 to −1.8	0.981–0.998	2 mol l^−1^ HOAc or 2 mol l^−1^ HCl
Ferromanganese (hydrogentic‐hydrothermal)	27–32	17.6–29.8	1270–35200	−14.4 to 0.5	0.986–1.000	2 mol l^−1^ HOAc or 2 mol l^−1^ HCl
Ferromanganese (hydrothermal)	32	30.3–32.9	3710–1188000	−1.7 to 0.9	0.998–1.001	2 mol l^−1^ HOAc or 2 mol l^−1^ HCl

The data of RS and RRS are from this study and Pogge von Strandmann *et al*. (2019a), the data of Greenland river suspended load are from Wimpenny *et al*. (2010b), the data of Dryadbreen glacial sediment are from Hindshaw *et al*. (2019), the data of Hawaii soil are from Li *et al*. (2020) and the data of Ferromanganese are from Chan and Hein (2007).

## Conclusions

(1) Based on a series of experiments exploring the effects of temperature, leaching time, reagent/solid ratio and reagent strength, we propose an optimised method for selectively leaching Li isotopes from oxides using 0.04 mol l^−1^ HH in 25% *v/v* HOAc with a reagent/solid ratio of 40 ml g^−1^ at room temperature (20 ± 2 °C) for 1 h. This leaching method applied on five solids in this study, including a fresh basalt, two weathered basalts, a Yellow River sediment and a shale, represents a balance between maximising the leaching of secondary oxides while minimising the influence of clays or primary minerals and is therefore not designed to quantitatively remove all Li associated with the oxide phases. It must be noted that the applicability of the leaching method may be somewhat altered by the origin and composition of the bulk sediment, and it is therefore always necessary to determine the elemental composition of each leachate.
(2) In full sequential leachates of silicates (i.e., selective leaching of the exchangeable, carbonate, oxide and silicate phases), the K concentrations appear to be a good indicator of exchangeable phases. If the silicates contain more than 5% carbonate, it is necessary to remove carbonate before leaching the oxide phase. Carbonates may also contain some Mn, meaning that Mn concentrations are not a reliable indicator of oxide or silicate phases. Our results also suggest that more than 95% of Fe and Al can be in silicates (clays and primary minerals), rather than in oxides, which demonstrates that there are no major elements exclusively found in oxides. However, Mg appears to be a good indicator of contributions from clays and primary silicate minerals because virtually no Mg is contained in oxides. Thus, combining Mg concentrations with Fe, Mn and Al would allow the targeted phases to better be distinguished.
(3) In the oxides leached by our optimal method, the Δ^7^Li_oxide‐solution_ ranges from −16.8‰ to −27.7‰, with Li concentrations that are intermediate between clays (with higher Li concentrations) and carbonates and the exchangeable fraction (with lower Li concentrations). As such, oxide minerals need to be considered as playing a potential role in fractionating Li isotopes during weathering at the Earth's surface.
(4) It is crucial that studies that utilise selective leaching to assess the compositions of specific phases within bulk sediments or soils also measure and publish the trace element data from each leachate. We have demonstrated that several frequently utilised leaching methods do not target single phases in isolation, and this impacts the Li budget of the leachates in some cases. Thus, trace element data are vital for ensuring the reliability and robustness of leachate Li isotopic compositions.

## Supporting information

Appendix S1. The mineralogy of solids.

Appendix S2. Reproducibility of elemental mass fractions and Li isotopes.

Appendix S3. Clarification of terminology.

Figure S1. Elemental ratios in oxide leachates from the different oxide leaching experimental trials.

Figure S2. Lithium mass fraction–Li isotopes diagrams and Mn–Mg–Fe ternary diagrams of oxide leaching trials of YR and SGR‐1b.

Figure S3. Flow chart of the full sequential extraction procedure.

Table S1. Mineral composition of RS from Jones *et al*. (2012).

Table S2. The procedure of cation‐exchange columns for Li purification.

Table S3. Element mass fractions and Li isotopes of all replicate samples in oxide leaching method experimental trials. This material is available from: http://onlinelibrary.wiley.com/doi/10.1111/ggr.12441/abstract (This link will take you to the article abstract).

## Data Availability

The data that support the findings of this study are available from the corresponding author upon reasonable request.
