# Supplementary material for: Experimental Investigation of Oxide Leaching Methods for Li Isotopes
Source: Geostand Geoanal Res. 2022 Jul 20;46(3):493–518. doi: 10.1111/ggr.12441 (PMC9544563; doi:10.1111/ggr.12441)
Supplement: Supplementary file 1 — Appendix S1. The mineralogy of solids. [file GGR-46-493-s006.pdf]

Geostandards and Geoanalytical Research (2022)

Online Supporting Information

Experimental Investigation of Oxide Leaching Methods for Li Isotopes

Chun-Yao **Liu***, Philip A.E. **Pogge von Strandmann**, Gary **Tarbuck** and David J. **Wilson**

* Corresponding author. e-mail: chunyao.liu.19@ucl.ac.uk

Appendix S1

The mineralogy of the solids

The mineral composition of RS is determined by Jones *et al*. (2012) (Table S1). The RRS is the reacted RS from an experiment in Pogge von Strandmann *et al*. (2019). So, in principle, the mineral composition of RRS should be similar to that of RS, and there was no resolvable difference reported by that study when using XRD and FTIR (Fourier-transform infrared spectroscopy) methods. According to PHREEQC calculation, it is likely that kaolinite, smectite, and iron oxyhydroxides formed during the interaction experiment (Pogge von Strandmann *et al*. 2019).

BCR-2, as was BCR-1, was collected from Bridal Veil Flow Quarry by U.S Geological Survey (Wilson 1998). BCR-1 is an aphanitic hypocrystalline basalt and andesine-trachybasalt (Flanagan 1967). Plagioclase, which is mainly acid plagioclase (Na-plagioclase) is the dominant mineral, the mafic minerals (pyroxenes, amphiboles, micas, olivine, melilite, magnetite, ilmenite, apatite) comprise around 40–45%. Among the mafic minerals, pyroxenes contribute more than magnesian olivine (around 1–5%), metallic opaque minerals (magnetite and ilmenite, less than 1–9%) (Hamilton 1963, Flanagan 1967).

SGR-1b, as also SGR-1, is collected from part of Mahogany Oil Shale Zone of the adjacent Piceance Creek Basin, Green River Formation, Western USA (Boak and Poole 2015). SGR-1 is a lacustrine sedimentary shale. According to the mineralogy of the shales near the site of SGR-1 in Green River formation, quartz, feldspar, K-feldspar, albite, dolomite and calcite comprise around 10–30%, 20–35%, 8–25%, 3–16%, 8–18%, and 6–10%, respectively. Clay minerals and organic matter comprise around 10–30% and 3–10%, respectively (Boak and Poole 2015).

YR is a sediment from the Yellow River estuary, to which loess from the Chinese Loess Plateau

contributes more than 90% of the minerals (Zhang *et al.* 1990). According to the mineral composition of sediments near the Yellow River estuary, the YR should contain around 40% quartz, 7% potassium feldspar, 15% plagioclase, 10% calcite and around 30% clay minerals (Yang *et al.* 2009, Wang and Jin 2017, Tian *et al.* 2021).

References

Boak J. and Poole S. (2015)
Mineralogy of the Green River Formation in the Piceance Creek Basin, Colorado. **In: Smith M.E. and Carroll A.R. (eds), Stratigraphy and paleolimnology of the Green River Formation (western USA). Springer**, 183–209.

Flanagan F.J. (1967)
U.S. Geological Survey silicate rock standards. **Geochimica et Cosmochimica Acta, 31**, 289–308.

Hamilton B.W. (1963)
Columbia River Basalt in the Riggins quadrangle, western Idaho. **Bulletin, 1114**, L1–L37.

Jones M.T., Pearce C.R. and Oelkers E.H. (2012)
An experimental study of the interaction of basaltic riverine particulate material and seawater. **Geochimica et Cosmochimica Acta, 77**, 108–120.

Pogge von Strandmann P.A.E., Fraser W.T., Hammond S.J., Tarbuck G., Wood I.G., Oelkers E.H. and Murphy M.J. (2019)
Experimental determination of Li isotope behaviour during basalt weathering. **Chemical Geology, 517**, 34–43.

Tian S., Li Z., Wang Z., Jiang E., Wang W. and Sun M. (2021)
Mineral composition and particle size distribution of river sediment and loess in the middle and lower Yellow River. **International Journal of Sediment Research, 36**, 392–400.

Wang Y. and Jin B. (2017)

Comparative analysis of carbonates in sediments of the Yellow River and the Haihe River estuaries. **Marine Sciences, 41**, 94–104. (in Chinese)

Wilson S. (1998)
United States Geological Survey certificate of analysis: Basalt, Columbia River, BCR-2. **United States Geological Survey report**.

Yang Z., Wang H. and Qiao S. (2009)
Carbonate minerals in estuary sediments of the Changjiang (Yangtze River) and Huanghe (Yellow River): The content, morphology, and influential factors. **Oceanologia et Limnologia Sinica, 40**, 674–681. (in Chinese)

Zhang J., Huang W.W. and Shi M.C. (1990)
Huanghe (Yellow River) and its estuary: Sediment origin, transport and deposition. **Journal of Hydrology, 120**, 203–223.
