# Supplementary material for: Experimental Investigation of Oxide Leaching Methods for Li Isotopes
Source: Geostand Geoanal Res. 2022 Jul 20;46(3):493–518. doi: 10.1111/ggr.12441 (PMC9544563; doi:10.1111/ggr.12441)
Supplement: Supplementary file 2 — Appendix S2. Reproducibility of elemental mass fractions and Li isotopes. [file GGR-46-493-s005.pdf]

Geostandards and Geoanalytical Research (2022)

Online Supporting Information

Experimental Investigation of Oxide Leaching Methods for Li Isotopes

Chun-Yao **Liu***, Philip A.E. **Pogge von Strandmann**, Gary **Tarbuck** and David J. **Wilson**

* Corresponding author. e-mail: chunyao.liu.19@ucl.ac.uk

Appendix S2

Reproducibility of elemental mass fractions and Li isotopes

The reproducibility of the elemental mass fractions in the oxide leaching experimental trials are shown as RSD (relative standard deviation) in Table 4. For the Tessier oxide leaching experiments, the RSD of replicate samples is generally better than 15% and most RSD (285 out of 320) are better than 10%. Among different elements, the poorest reproducibility is seen for K mass fractions; for example, RRS after 6 h of leaching at high temperature and SGR-1b after 6 h of leaching at room temperature have an RSD > 30% for K. Generally, the Tessier oxide leaching experimental trials have better reproducibility of elemental mass fractions than the strong oxide leaching method. The RSD for the weak oxide leaching method is particularly high, ranging from 2.8% to 41.0%, especially for RS, RRS and BCR-2.

The reproducibility of δ^7Li values are indicated by the standard deviation (*s*) of δ^7Li in full procedural replicate samples (Table 4). All the *s* of δ^7Li is better than ± 1.0‰. Half of the *s* are better than 0.4‰ and 75% of the *s* are better than ± 0.6‰. Note that the long-term external reproducibility based on reference materials such as seawater and totally dissolved rock reference materials such as BCR-2, G-2, PCC-1 and SGR-1b is ± 0.4‰ (2*s*) (Pogge von Strandmann *et al.* 2019), Given that the reproducibility of the chemical purification and isotope analysis is consistent across multiple different matrices, it seems likely that the slightly poorer reproducibility of the oxide leachate replicates arises from the leaching method itself (e.g., slight changes in efficiency, or sample heterogeneity) rather than from the isotope purification or analysis directly. Although the application of sequential leaching appears to slightly degrade the reproducibility of Li isotope measurements, the reproducibility among replicates is fairly good overall.

Reference

Pogge von Strandmann P.A.E., Fraser W.T., Hammond S.J., Tarbuck G., Wood I.G., Oelkers E.H. and Murphy M.J. (2019)
Experimental determination of Li isotope behaviour during basalt weathering. **Chemical Geology, 517**, 34–43.
