# Supplementary material for: Experimental Investigation of Oxide Leaching Methods for Li Isotopes
Source: Geostand Geoanal Res. 2022 Jul 20;46(3):493–518. doi: 10.1111/ggr.12441 (PMC9544563; doi:10.1111/ggr.12441)
Supplement: Supplementary file 3 — Appendix S3. Clarification of terminology. [file GGR-46-493-s008.pdf]

Geostandards and Geoanalytical Research (2022)
Online Supporting Information

Experimental Investigation of Oxide Leaching Methods for Li Isotopes

Chun-Yao **Liu***, Philip A.E. **Pogge von Strandmann**, Gary **Tarbuck** and David J. **Wilson**
* Corresponding author. e-mail: chunyao.liu.19@ucl.ac.uk

Appendix S3

Clarification of terminology

According to the International Vocabulary of Metrology, "concentration" refers to the mass per volume, while "mass fraction" refers to the mass per mass. So as to provide a clarification in the section '*Lithium isotopes and elemental concentration analysis*', the elemental mass fractions in an extraction (a phase or "oxide" in a trial) from the total solid are reported as measured elemental concentrations in leachates normalised to the total mass of leached solid.

The exchangeable Li is referred to the Li, which is adsorbed (exchanged) on the negatively charged external and internal surfaces of the clays.

The carbonate Li is referred to the Li in the carbonate. In principle, Li could substitute for Ca or Mg. In addition, Li could be in some interstitial positions (Marriott *et al*. 2004).

Oxide(s) include primary oxides and secondary oxides. In this study, if there is no special explanation of context, the single word oxide refers to secondary oxides. In this study, we mainly consider the common secondary oxides (i.e., Fe/Mn/Al secondary oxides). Based on our data, as discussed in the section entitled '*Comparison of Tessier, weak and strong oxide leaching methods*', the less-structured oxides and Mn oxides, and "pH-sensitive" oxides (i.e., gibbsite) are regarded as "readily-leached" oxides. The Fe secondary oxides are considered to be harder to leach.

Clay(s) in this study refers to those that form during low-temperature weathering on the Earth's surface. In principle, the clays that form during hydrothermal alteration are not included. In sedimentary rocks, the secondary clay refers to that which forms during weathering of sedimentary rocks instead of the formation of sedimentary rocks. But in practice, these different clays were hard to distinguish by chemical extraction in this study.

(Silicate) residue refers to the remaining solids after extraction of exchangeable, carbonate, oxide and clay. For the five solids in this study, the residue is dominated by silicate minerals.

The bulk refers to the whole solid. To clarify, there are two data sources of the element mass fraction in a bulk solid. One is from published papers, which could have been measured by AAS (atomic absorption spectrometry), ICP-AES/ICP-MS after total dissolution, scanning electron microscopy (SEM), etc. The other source is calculated from the full sequential leaching in this study. These two types of bulk data are distinguished by the context.

The Tessier (oxide) leaching/Tessier (oxide) extraction/Tessier leachates refers to the oxide extraction or the oxide leachates by the method based on Tessier *et al*. (1979), which is 0.04 mol l^{-1} HH in 25% *v/v* HOAc.

The weak(er) (oxide) leaching/weak (oxide) extraction/weak(er) leachates refers to the oxide extraction or the oxide leachates by the method based on Hindshaw *et al.* (2018), which is 0.005 mol l^{-1} HH in 2.6 mol l^{-1} HOAc with reagent/solid ratio of 3.125 ml g^{-1} at room temperature for 1 h.

The strong(er) (oxide) leaching/strong (oxide) extraction/strong(er) leachates refers to the oxide extraction or the oxide leachates by the method based on the Community Bureau of Reference (BCR, Rauret *et al.* 1999, Li *et al.* 2020), which is 0.5 mol l^{-1} HH in 0.05 mol l^{-1}

HNO_3 (pH = 1.5) with reagent/solid ratio of 40 ml g^{-1} at room temperature for 16 h.

References

Hindshaw R.S., Tosca R., Goût T.L., Farnan I., Tosca N.J. and Tipper E.T. (2019)
Experimental constraints on Li isotope fractionation during clay formation. **Geochimica et Cosmochimica Acta, 250**, 219–237.

Li W., Liu X.-M. and Chadwick O.A. (2020)
Lithium isotope behavior in Hawaiian regoliths: Soil-atmosphere-biosphere exchanges. **Geochimica et Cosmochimica Acta, 285**, 175–192.

Marriott C.S., Henderson G.M., Crompton R., Staubwasser M. and Shaw S. (2004)
Effect of mineralogy, salinity, and temperature on Li/Ca and Li isotope composition of calcium carbonate. **Chemical Geology, 212**, 5–15.

Rauret G., López-Sánchez J.F., Sahuquillo A., Rubio R., Davidson C., Ure A. and Quevauviller Ph. (1999)
Improvement of the BCR three step sequential extraction procedure prior to the certification of new sediment and soil reference materials. **Journal of Environmental Monitoring, 1**, 57–61.

Tessier A., Campbell P.G.C. and Bisson M. (1979)
Sequential extraction procedure for the speciation of particulate trace metals. **Analytical Chemistry, 51**, 844–851.
