# Supplementary material for: Experimental Investigation of Oxide Leaching Methods for Li Isotopes
Source: Geostand Geoanal Res. 2022 Jul 20;46(3):493–518. doi: 10.1111/ggr.12441 (PMC9544563; doi:10.1111/ggr.12441)

Geostandards and Geoanalytical Research (2022)
Online Supporting Information

Experimental Investigation of Oxide Leaching Methods for Li Isotopes

Chun-Yao **Liu***, Philip A.E. **Pogge von Strandmann**, Gary T**arbuck** and David J. **Wilson**
* Corresponding author. e-mail: chunyao.liu.19@ucl.ac.uk

Figure S1. Elemental ratios in oxide leachates from the different oxide leaching experimental trials.

See text for details of the different leaching methods.
The range bars represent RSD of the replicates.

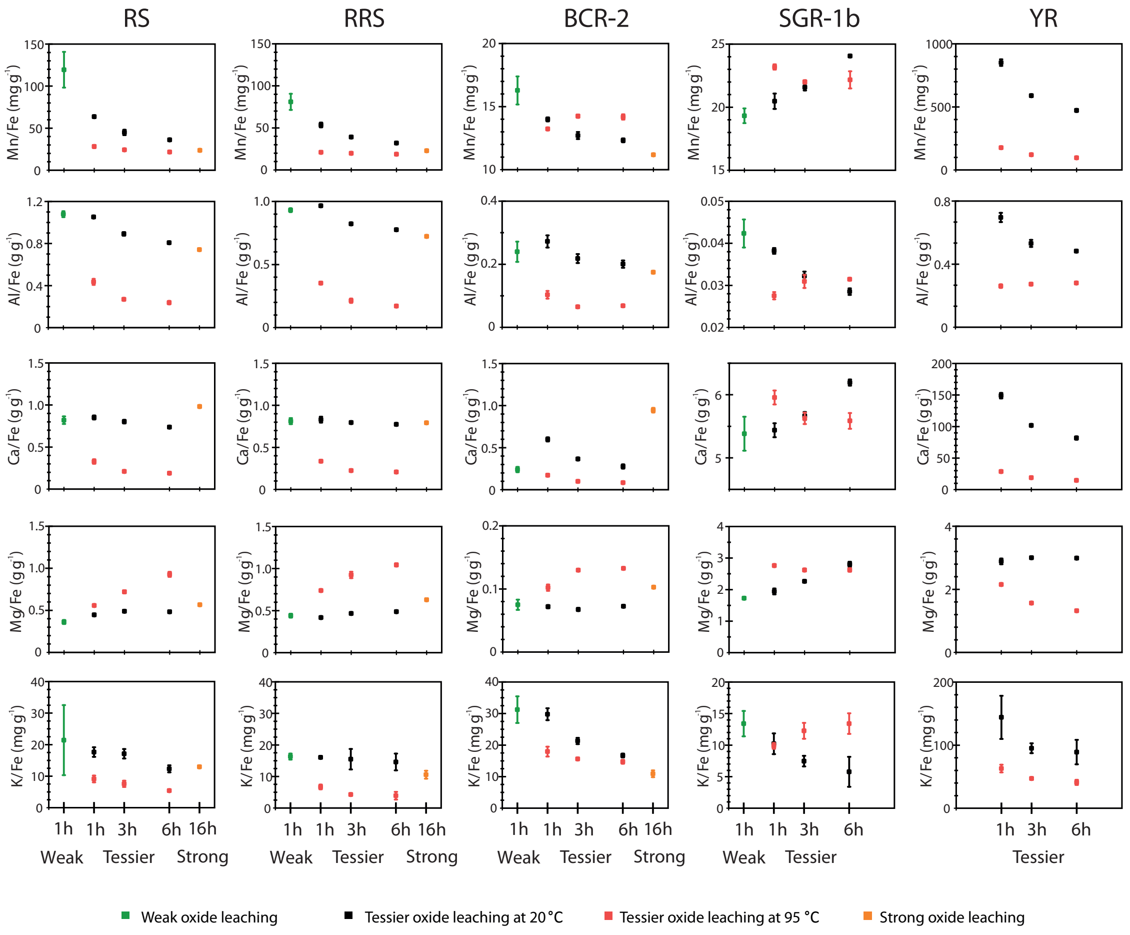

Supplement: Supplementary file 4 — Figure S1. Elemental ratios in oxide leachates from the different oxide leaching experimental trials. [file GGR-46-493-s007.pdf]
