# Supplementary material for: Experimental Investigation of Oxide Leaching Methods for Li Isotopes
Source: Geostand Geoanal Res. 2022 Jul 20;46(3):493–518. doi: 10.1111/ggr.12441 (PMC9544563; doi:10.1111/ggr.12441)

Geostandards and Geoanalytical Research (2022)
Online Supporting Information

Experimental Investigation of Oxide Leaching Methods for Li Isotopes

Chun-Yao **Liu***, Philip A.E. **Pogge von Strandmann**, Gary T**arbuck** and David J. **Wilson**
* Corresponding author. e-mail: chunyao.liu.19@ucl.ac.uk

Figure S2. Lithium mass fraction - Li isotopes diagrams and Mn-Mg-Fe ternary diagrams of oxide leaching trials of YR and SGR-1b.

The data of bulk solid is from Gladney and Roelandts (1988), Pogge von Strandmann *et al.* (2019) and Hindshaw *et al.* (2019).

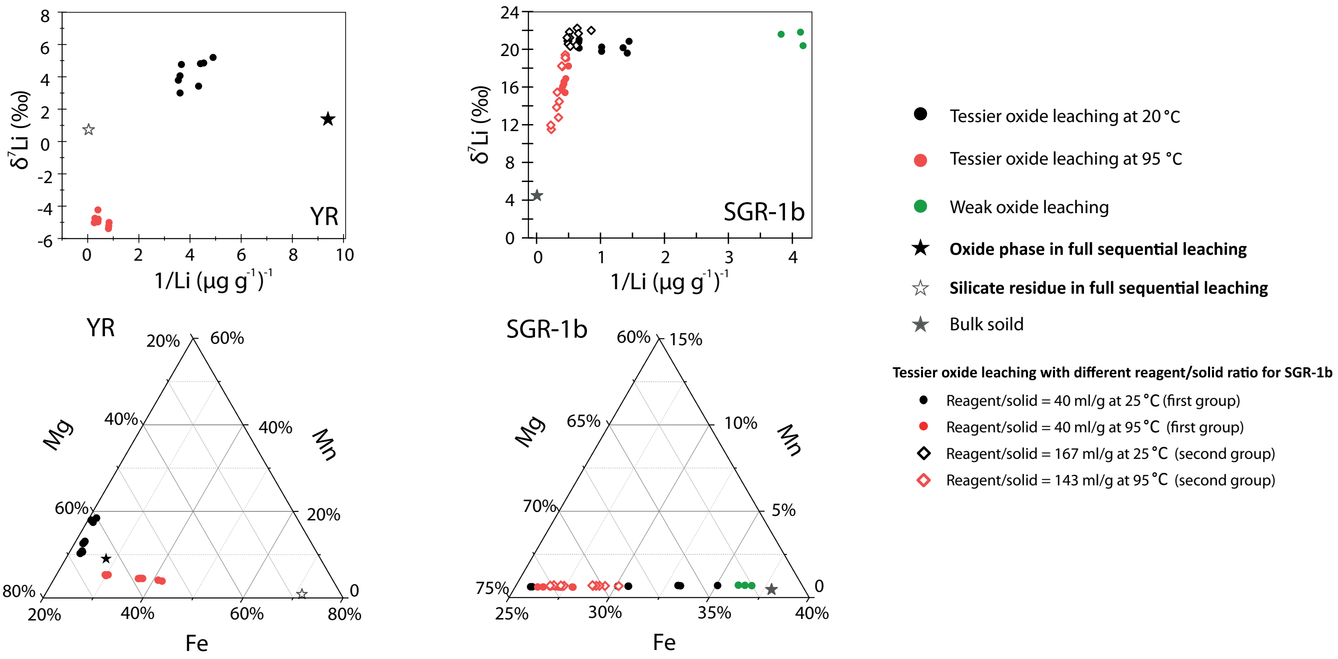

Supplement: Supplementary file 5 — Figure S2. Lithium mass fraction–Li isotopes diagrams and Mn–Mg–Fe ternary diagrams of oxide leaching trials of YR and SGR‐1b. [file GGR-46-493-s004.pdf]
