# Supplementary material for: Experimental Investigation of Oxide Leaching Methods for Li Isotopes
Source: Geostand Geoanal Res. 2022 Jul 20;46(3):493–518. doi: 10.1111/ggr.12441 (PMC9544563; doi:10.1111/ggr.12441)

**Geostandards and Geoanalytical Research (2022)**
**Online Supporting Information**

**Experimental Investigation of Oxide Leaching Methods for Li Isotopes**

Chun-Yao **Liu***, Philip A.E. **Pogge von Strandmann**, Gary T**arbuck** and David J. **Wilson**
* Corresponding author. e-mail: chunyao.liu.19@ucl.ac.uk

**Figure S3.** Flow chart of the full sequential extraction procedure.

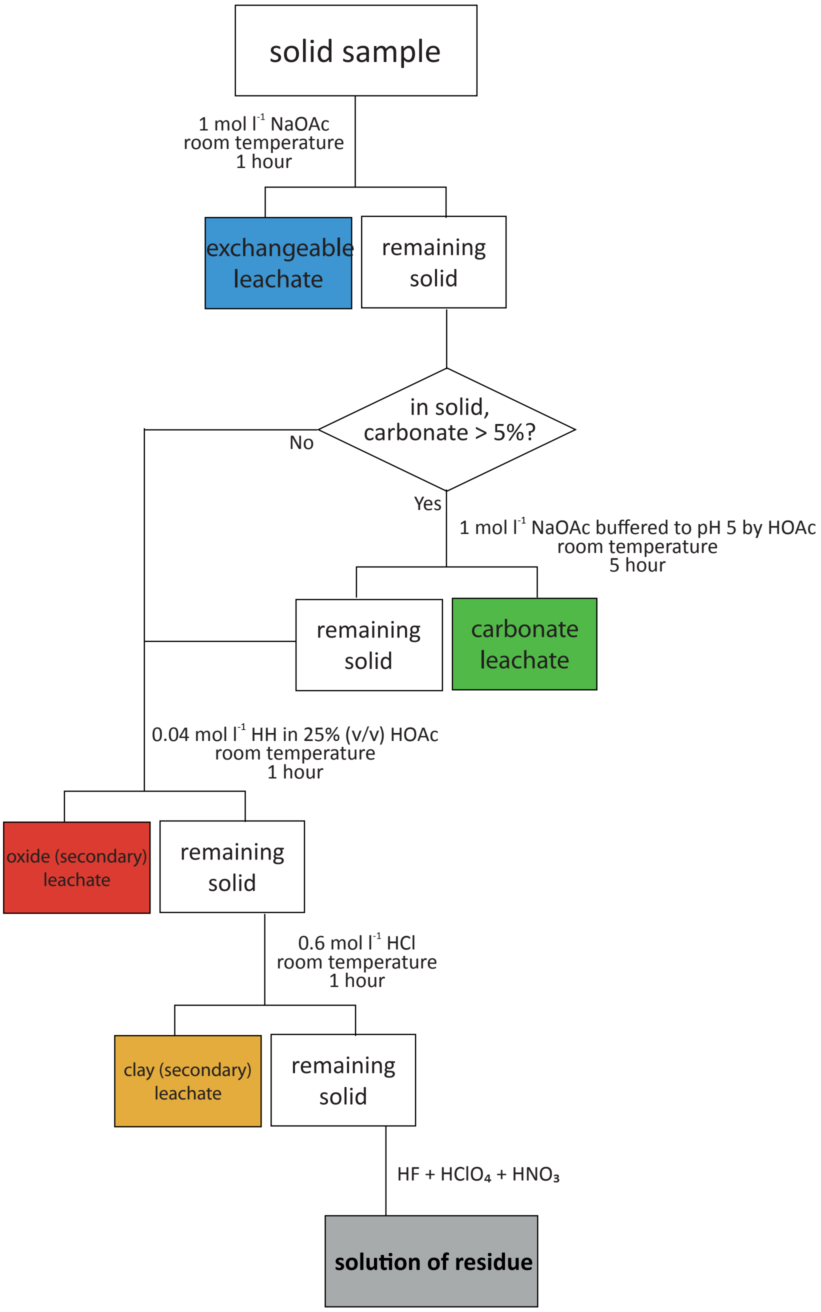

Supplement: Supplementary file 6 — Figure S3. Flow chart of the full sequential extraction procedure. [file GGR-46-493-s002.pdf]
