# Supplementary material for: Experimental Investigation of Oxide Leaching Methods for Li Isotopes
Source: Geostand Geoanal Res. 2022 Jul 20;46(3):493–518. doi: 10.1111/ggr.12441 (PMC9544563; doi:10.1111/ggr.12441)
Supplement: Supplementary file 7 — Table S1. Mineral composition of RS from Jones et al. (2012). [file GGR-46-493-s001.docx]

Geostandards and Geoanalytical Research (2022)

**Online Supporting Information**

Experimental Investigation of Oxide Leaching Methods for Li Isotopes

Chun-Yao **Liu***, Philip A.E. **Pogge von Strandmann**, Gary **Tarbuck** and David J. **Wilson**

* Corresponding author. e-mail: chunyao.liu.19@ucl.ac.uk

Table S1.

Mineral composition of RS from Jones *et al*. (2012)

| **Mineral** | **Composition** | |
| --- | --- | --- |
|  | **(%)** |  |
|  |  |  |
| Microcrystalline | 50.25 |  |
| Volcanic glass | 18.23 |  |
| Ca feldspar | 10.84 |  |
| Olivine | 7.39 |  |
| Fe-Ti oxides | 4.93 |  |
| K feldspar | 2.46 |  |
| Quartz | 1.48 |  |
| Calcite | 4.43 |  |
