# Supplementary material for: Experimental Investigation of Oxide Leaching Methods for Li Isotopes
Source: Geostand Geoanal Res. 2022 Jul 20;46(3):493–518. doi: 10.1111/ggr.12441 (PMC9544563; doi:10.1111/ggr.12441)
Supplement: Supplementary file 8 — Table S2. The procedure of cation‐exchange columns for Li purification. [file GGR-46-493-s009.docx]

Geostandards and Geoanalytical Research (2022)

**Online Supporting Information**

Experimental Investigation of Oxide Leaching Methods for Li Isotopes

Chun-Yao **Liu***, Philip A.E. **Pogge von Strandmann**, Gary **Tarbuck** and David J. **Wilson**

* Corresponding author. e-mail: chunyao.liu.19@ucl.ac.uk

Table S2.

The procedure of cation-exchange columns for Li purification

| The first column | |
| --- | --- |
| 3.2 mm (radius), 8.5 cm (height) resin. | |
| Preconditioning | 4 ml 0.2 mol l^-1^ HCl |
| Load sample | 0.1 ml 0.2 mol l^-1^ HCl |
| Elution | 17.5 ml 0.2 mol l^-1^ HCl |
| Split pre-collection | 1 ml 0.2 mol l^-1^ HCl |
| Collection | 20 ml 0.2 mol l^-1^ HCl |
| Split after-collection | 1 ml 0.2 mol l^-1^ HCl |
| Cleaning | 6 mol l^-1^ HCl and ultrapure water |
| The second column | |
| ∼ 1 mm (radius), 5 cm (height) resin. | |
| Preconditioning | 1 ml 0.2 mol l^-1^ HCl |
| Load sample | 0.1 ml 0.2 mol l^-1^ HCl |
| Elution | 4.9 ml 0.2 mol l^-1^ HCl |
| Split pre-collection | 1 ml 0.2 mol l^-1^ HCl |
| Collection | 12 ml 0.2 mol l^-1^ HCl |
| Split after-collection | 1 ml 0.2 mol l^-1^ HCl |
| Cleaning | 6 mol l^-1^ HCl and ultrapure water |
